# Changes in asset-based wealth across the life course in birth cohorts from five low- and middle-income countries

**DOI:** 10.1016/j.ssmph.2021.100976

**Published:** 2021-11-20

**Authors:** Jithin Sam Varghese, Linda S. Adair, Shivani A. Patel, Sonny Agustin Bechayda, Santosh K. Bhargava, Delia B. Carba, Bernardo L. Horta, Natalia P. Lima, Reynaldo Martorell, Ana M.B. Menezes, Shane A. Norris, Linda M. Richter, Manuel Ramirez-Zea, Harshpal Singh Sachdev, Fernando C. Wehrmeister, Aryeh D. Stein, for the COHORTS group

**Affiliations:** a Nutrition and Health Sciences Program, Laney Graduate School, Emory University, Atlanta, GA, USA; b Department of Nutrition, Gillings School of Global Public Health, University of North Carolina at Chapel Hill, Chapel Hill, NC, USA; c Hubert Department of Global Health, Rollins School of Public Health, Emory University, Atlanta, GA, USA; d USC-Office of Population Studies Foundation, Inc, University of San Carlos, Cebu City, Philippines; e Department of Anthropology, Sociology, and History, University of San Carlos, Cebu City, Philippines; f Founder, New Delhi Birth Cohort, New Delhi, India; g Postgraduate Program in Epidemiology, Federal University of Pelotas, Pelotas, Brazil; h Postgraduate Program in Health and Behavior, Catholic University of Pelotas, Pelotas, Brazil; i SAMRC Developmental Pathways for Health Research Unit, University of the Witwatersrand, Johannesburg, South Africa; j DSI-NRF Centre of Excellence in Human Development, University of the Witwatersrand, Johannesburg, South Africa; k INCAP Research Center for the Prevention of Chronic Diseases (CIIPEC), Institute of Nutrition of Central America and Panama (INCAP), Guatemala City, Guatemala; l Sitaram Bhartia Institute of Science and Research, New Delhi, India

**Keywords:** CLHNS, Cebu Longitudinal Health and Nutrition Survey, COHORTS, Consortium On Health Orientated Research in Transitioning Societies, EFA, Exploratory Factor Analysis, INCAP, Institute of Nutrition for Central America and Panama, LMIC, Low- and middle-income countries, MCA, Multiple Correspondence Analysis, NDBC, New Delhi Birth Cohort, PCA, Principal Component Analysis, SD, standard deviation, SEP, Socio-economic position, Wealth index, Life course epidemiology, Social mobility

## Abstract

**Background:**

Temporally-harmonized asset-based measures of wealth can be used to study the association of life-course wealth exposures in the same scale with health outcomes in low- and middle-income countries (LMICs). The within-individual longitudinal stability of asset-based indices of wealth in LMICs is poorly understood.

**Methods:**

Using data from five birth cohorts from three continents, we developed temporally-harmonized asset indices over the life course through polychoric principal component analysis of a common set of assets collected consistently over time (18 years in Brazil to 50 years in Guatemala). For each cohort, we compared the harmonized index to cross-sectional indices created using more comprehensive asset measures using rank correlations. We evaluated the rank correlation of the harmonized index in early life and adulthood with maternal schooling and own attained schooling, respectively.

**Results:**

Temporally-harmonized asset indices developed from a consistently-collected set of assets (range: 10 in South Africa to 30 in Philippines) suggested that mean wealth improved over time for all birth cohorts. Cross-sectional indices created separately for each study wave were correlated with the harmonized index for all cohorts (Brazil: r = 0.78 to 0.96; Guatemala: r = 0.81 to 0.95; India: 0.75 to 0.93; Philippines: r = 0.92 to 0.99; South Africa: r = 0.84 to 0.96). Maternal schooling (r = 0.15 to 0.56) and attained schooling (r = 0.23 to 0.53) were positively correlated with the harmonized asset index in childhood and adulthood respectively.

**Conclusions:**

Temporally-harmonized asset indices displayed coherence with cross-sectional indices as well as construct validity with schooling.

## Highlights

• Temporally-harmonized asset indices are useful to assess relative importance of wealth at different life stages with health on the same scale.
• Harmonized indices using a subset of assets were correlated with cross-sectional asset indices using all available assets in five LMIC birth cohorts.
• Harmonized indices displayed construct validity, as demonstrated by its correlation with schooling.
• Harmonized indices were robust to alternate specifications such as shorter lists of assets, study years, and factor extraction procedures.

## 1 Introduction

Low- and middle-income countries (LMIC) have experienced a rise in material living standards from investments in human capital and rise in global trade (World Development Indicat). This economic transition parallels demographic and epidemiological transitions wherein the burden of diseases has shifted from infectious diseases and maternal, child and newborn illnesses to non-communicable diseases (Jaacks et al., 2019; Popkin, 2015; NCD Risk Factor Collaboration, 2017; Yusuf et al., 2001). Previous research has reported that these transitions tend to percolate down from those belonging to high socio-economic position to low socio-economic position in LMICs (Templin et al., 2019).

Wealth, a dimension of material wellbeing, is an indicator of socio-economic position (SEP) in societies that are vulnerable to income shocks and unforeseen expenditures (Howe et al., 2008; Krieger et al., 1997). Asset indices are useful proxy measures of wealth in LMICs where ownership of household items, high-quality housing and public services are not universal (Johnston & Abreu, 2016). Asset indices are reflective of long-run cumulative economic status and are correlated with expenditures on non-food items and household public goods in the absence of transitory shocks to spending (Filmer & Scott, 2012).

The role of longitudinal changes in individual earnings or household wealth on health over the life course in LMICs is less understood relative to high-income countries, primarily due to unavailability of longitudinal data. In LMICs, populations have experienced substantial changes but with persisting inequalities over the past five decades (Dorling et al., 2007; Ward & Viner, 2017). Existing literature on this topic has relied primarily on cross-sectional survey data that describe aggregate trends in household wealth using temporally valid asset indices, and do not directly quantify individual impacts of household level changes in wealth over time (Booysen et al., 2008; Rutstein & Staveteig, 2013; Sahn & Stifel, 2000; Smits & Steendijk, 2014; Woolard et al., 2021). Previous methodological advances for making household wealth comparable over time and geography include the International Wealth Index (IWI), the Comparative Wealth Index (CWI) and the Absolute Wealth Estimate (AWE). However, their applicability has to date been restricted largely to serial cross-sectional studies. The IWI uses a common set of seven consumer durables, three housing characteristics and two public utilities in 165 cross-sectional surveys from 97 countries. The CWI was based on a reference Demographic and Health survey. The AWE was based on cross-sectional asset indices, national estimates of gross domestic product per capita and income inequality, expressed in 2011-constant dollars. While providing comparability over time and geography, due to the nature of the data sources, these measures do not permit exploration of long-term household-level changes in wealth that could aid in understanding the importance of life stage (such as early life, adolescence and early adulthood) and dimensions of SEP (wealth, schooling, employment) for later-life health outcomes (such as cardiovascular and metabolic disease) and wellbeing at an individual level (Howe et al., 2012). Additionally, such studies could contribute towards understanding the role of life course wealth in health disparities that are present in adulthood. Given the pace of the economic, demographic and epidemiological transitions experienced by LMICs, it is important to study wealth mobility over the life course and how it is associated with health outcomes later in life. Under assumptions of assets as public goods, household wealth reflecting an individual's wealth, similar asset loadings over time, and empirical demonstration of rank similar to standard cross-sectional approach, we may estimate these associations even if individuals were to change households (such as following adoption, migration or marriage).

Our objective was to develop a temporally-harmonized asset index over the life course for LMIC birth cohorts and assess its construct validity (i.e., the extent to which it ranks individuals on their socio-economic position) as well as robustness (i.e., the extent to which results are similar across alternate specifications of assets, survey years and factor extraction procedures) (Rutstein & Staveteig, 2013; Smits & Steendijk, 2014). Such an index would allow researchers to compare wealth at different stages over the life course on the same measurement scale. The birth cohorts are part of the Consortium for Health Oriented Research from Transitioning Societies (COHORTS) collaborative (Richter et al., 2012). The cohorts are from five countries across three continents that have experienced different trajectories of economic development (Supplementary Fig. 1).

We compared the temporally-harmonized index created in our study for each cohort with cross-sectional and regional (urban, rural) indices as per standard practice in epidemiological studies (Filmer & Pritchett, 2001). We assessed if the harmonized index displays construct validity using maternal schooling and attained schooling. We also assessed the extent of generalizing findings to similar settings by assessing the robustness of the temporally-harmonized index to alternate specifications derived from including specific assets (shorter data collection instruments) and years of data collection (unmeasured effect modification by age or period effects) or different factor extraction procedures.

## 2 Methods

### 2.1 Study population

We used all available information on assets collected over the life course for each of 5 birth cohorts - Brazil (Pelotas 1993), Guatemala (INCAP Longitudinal Study), India (New Delhi Birth Cohort), Philippines (Cebu Longitudinal Health and Nutrition Survey) and South Africa (Birth to Twenty plus cohort). The cohorts are representative of urban areas (Brazil, India, and South Africa) or a mix of urban and rural areas (Guatemala, Philippines) in these countries. We present a detailed description of study waves used for each cohort in Table 1. For the INCAP Longitudinal Study cohort (from Guatemala), which includes multiple individuals from the same household, we conducted our analysis at the household level. All other cohorts consisted of only one participant per household.

**Table 1:** Percentage of birth cohort with valid asset data at each study wave.

	Pelotas 1993 (Brazil)^a^		INCAP (Guatemala) b		NDBC (India)^c^		CLHNS (Philippines)		Birth to Twenty plus (South Africa)	
	Start of study	N	Start of study	N	Start of study	N	Start of study	N	Start of study	N
	1993	5249	1969–77	2392	1969–72	8181	1983–84	3080	1990	3273
	Age at wave	%	Age at wave	%	Age at wave	%	Age at wave	%	Age at wave	%
1	3–4	24.2%	0–5	67.0%	27–33	18.7%	0	100%	0–2	85.9%
2	11–12	84.3%	0–7	92.5%	34–40	14.0%	7–8	73.5%	7–8	41.1%
3	13–14	82.7%	10–25	56.9%	40–47	9.7%	12–13	71.0%	12–13	44.1%
4	18	78.2%	19–34	35.7%	44–51	10.3%	15–16	67.6%	16–17	46.2%
5	22	72.6%	25–40	44.0%			18–19	65.4%	22–23	50.0%
6			37–55	48.6%			21–22	61.2%	27–28	42.6%
7			40–57	52.9%			25–26	55.5%		
8							33–36	43.1%		

a Age 3–4 was a systematic sub-sample.

b Village enumeration collected data on assets and housing characteristics only for those who lived there.

c Early life waves did not collect asset data to include in this analysis.

The 1993 Pelotas (Brazil) cohort includes 5249 children born in the 1993 calendar year in the city of Pelotas. Study visits in childhood systematically targeted subsamples of cohort members residing in the city (Goncalves et al., 2018). The Institute of Nutrition for Central America and Panama (INCAP) conducted a nutrition supplementation cluster-randomized trial to study the role of early life protein and energy supplementation on growth and human capital from 1969 to 1977 in Guatemala (Stein et al., 2008). The trial was conducted in four villages of Department of El Progreso and the sample consisted of 2392 rural ladino (non-indigenous) residents of the study villages born between 1962 and 1977. The New Delhi Birth Cohort (India) consists of 8181 singleton births to married women in 1969–72 (Vasan et al., 2018). The Cebu Longitudinal Health and Nutrition Survey (Philippines) consisted of all pregnant women from a single-stage cluster-sample of 17 urban and 16 rural barangays in Metro Cebu in 1983 (Adair et al., 2011). Among the 3327 women interviewed at baseline, the sample consisted of 3080 singleton and 26 multiple births followed-up during subsequent waves. The Birth to Twenty plus study (South Africa) consists of 3273 singletons who were residents of Soweto-Johannesburg (urban) born during a 7-week enrollment period in 1990 (Richter et al., 2007).

All participants (or their parent, as appropriate) provided written informed consent prior to participation at each study wave. We obtained ethical approval from the Institutional Review Board of Emory University (Protocol 95960) for this analysis.

### 2.2 Indicators of wealth

Information on assets and housing characteristics (such as building material and type of toilet) were collected over the life course until they became irrelevant or negligible in value. New assets were added over time to reflect the changing pattern of wealth-defining asset ownership in each society. Assets which were no longer relevant were not collected, so the number and type of assets were variable across study waves. Detailed information on asset availability for each cohort is presented in Supplementary Tables 1A–E.

We included ownership (yes/no) of assets such as television, radio and washing machine as well as house ownership and electricity provision. We characterized housing by building material and type of toilet into ordinal variables (Low, Medium, High) based on site-specific definitions. We defined crowding as number of bedrooms per household member for Brazil, Guatemala, and Philippines such that a higher number represents greater wealth (Wall & Johnston, 2008). This is unlike the typical definition for crowding, which is household members per room.

### 2.3 Statistical analysis

We conducted all analysis at the household level, separately by cohort. We compared early life characteristics of cohort participants by participation in study wave. For the temporally-harmonized index, we considered all assets that were collected across all waves or were at most missing in one wave only. The list of assets considered varied by cohort. Within a cohort, we imputed the value for a missing asset for a wave based on the preceding study wave for those households that participated in that wave. For households that did not participate in the preceding wave, we imputed the missing value with the cross-sectional mode.

We performed a principal component analysis (PCA) on the polychoric correlation matrix derived after pooling study waves for each cohort (Poirier et al., 2019). PCA is a statistical procedure which projects observed data into a set of orthogonal principal components such that the first component explains the most variance in the data. We extracted the first component as the harmonized index. Additional information on the analytic procedure is available in Supplementary Note 1. The polychoric correlation assumes a normally distributed latent variable that underlies an observed binary or ordinal variable. A harmonized index that was inversely weighted by the size of the analytic sample at each study wave was similar (r = 1.00; results not shown) to the unweighted harmonized index.

We visually assessed the harmonized index at each study wave for clumping (many households having the same value of the index). We also visually examined the index for truncation, whereby the index fails to differentiate heterogeneity in asset ownership across households/individuals at high or low levels of the index. To resolve these issues would require including assets that are able to differentiate such observations along the index (Smits & Steendijk, 2014). However, such assets were not available over the life course.

### 2.4 Validation of harmonized index

To examine how our benchmark harmonized index performed relative to standard practice, we assessed the Spearman rank correlation with separate cross-sectional indices constructed using the same set of assets. We also created cross-sectional indices by urban and rural residence of cohort members when relevant (Guatemala, Philippines). We conducted this analysis because there is an implicit assumption for the harmonized index that material goods have the same meaning over time for a cohort. We also assessed the rank correlation of the temporally-harmonized index with cross-sectional indices created using all available assets for each study wave after removing those displaying near zero variances (prevalence ratio >95:5). To examine the degree of similarity of asset loadings over time, we calculated the Tucker coefficient of congruence (phi; same: greater than 0.95, high: 0.90 to 0.95, moderate: 0.85 to 0.89) between the harmonized index and each cross-sectional asset index created using the same set of assets after deleting zero-variance assets.

Finally, among those who participated in adulthood waves, we assessed the correlation of maternal schooling (collected in early life) and the participant's own attained schooling (in adulthood) with the corresponding measures of the harmonized index.

### 2.5 Sensitivity analysis

We assessed if the asset index was sensitive to inclusion of specific assets or to factor extraction procedure. We report the rank correlation of our harmonized index with indices created after dropping assets and study waves as well as using an alternate correlation matrix (Pearson) with different factor extraction (Exploratory Factor Analysis, Multiple Correspondence Analysis) procedures. We categorized all ordinal variables (Low or Medium versus High) into binary variables for estimation of Pearson correlation matrix. We additionally categorized continuous variables (crowding >0.75 rooms per member set to one, otherwise zero) into binary variables for the Multiple Correspondence Analysis.

We carried out all analysis using R 3.6.1 and ‘psych’ package v1.9.12.

## 3 Results

Information on a consistent set of durable assets and housing characteristics were available for each of the five birth cohorts over their life course (Supplementary Tables 1A–E; range of included assets 10 in South Africa to 30 in Philippines). Ownership of assets varied over time. The extent of ownership of electronic goods and quality of housing characteristics increased over time in all cohorts. Comparison of early life characteristics between children in all recruited households and children in households where asset data were unavailable (because the child did not participate or died) suggested that they were similar in Brazil, India, Philippines and similar on most characteristics in Guatemala and South Africa (Supplementary Tables 2A–E). Those who did not provide asset data in Guatemala were more likely to be male and in South Africa were more likely to be of White or Indian ethnicity, relative to the original sample.

### 3.1 Harmonized index construction

The harmonized index explained 44.6%, 54.4%, 26.5%, 35.5% and 48.4% of the variance in the polychoric correlation matrix for the cohorts from Brazil, Guatemala, India, Philippines and South Africa, respectively (Table 2). Ownership of large electronic appliances such as television, refrigerator, microwave, air conditioner and computer, consistently contributed to high positive loadings, such that households that owned these assets had higher values of the asset index. Ownership of radio (in Brazil and Guatemala) and farm animals (poultry, cattle, other animals) in Philippines had negative loadings, such that over time the households that owned them had lower values of the asset index.

**Table 2:** Loadings on temporally-harmonized index for assets and housing characteristics by cohort.

	Pelotas 1993 (Brazil)	INCAP (Guatemala)	NDBC (India)	CLHNS (Philippines)	Birth to Twenty plus (South Africa)
*Variance explained by PC1 (%)*	44.6%	54.4%	26.5%	35.5%	48.4%
Rooms per person	0.32	0.44	0.29	0.33	–
Car	0.74	0.81	0.75	0.81	0.65
Computer	0.81	–	0.89	–	–
Duplex refrigerator	0.67	–	–	–	–
DVD player	0.77	–	–	–	–
Housekeeper	0.63	–	–	–	–
Radio	−0.19	−0.17	−0.24	–	0.48
Refrigerator	0.54	0.9	–	0.88	0.87
Television	0.84	0.94	0.02	0.75	0.81
Vacuum cleaner	0.77	–	–	–	–
Washing machine	0.77	–	0.66	–	0.83
Drinking water quality	0.62	0.81	0.12	0.53	0.64
Bicycle	–	0.59	0.35	0.36	–
Electricity	–	0.94	–	0.83	0.69
Two wheeler	–	0.72	0.03	–	–
Owns house	–	0.09	–	0.09	–
Sewing machine	–	0.46	–	–	–
Floor quality	–	0.87	–	–	–
Kitchen location	–	0.68	–	–	–
Roof quality	–	0.82	–	–	–
Sewage facility	–	0.81	–	–	–
Stove/Cooking fuel quality	–	0.84	–	0.82	–
Toilet quality	–	0.8	0.53	0.79	0.66
Wall quality	–	0.85	–	–	–
Air conditioner	–	–	0.89	0.80	–
Cable TV	–	–	−0.44	–	–
Cell phone	–	–	0.81	–	–
Cooler	–	–	−0.53	–	–
Dish TV	–	–	0.83	–	–
Mixer grinder	–	–	0.57	–	–
Telephone	–	–	0.01	–	0.43
Sharing of drinking water source	–	–	−0.01	–	–
General water	–	–	0.07	–	–
Sharing of general water source	–	–	−0.14	–	–
Poultry	–	–	–	−0.18	–
Electric fan	–	–	–	0.78	–
Electric iron	–	–	–	0.87	–
Jeepny	–	–	–	0.65	–
Living room set	–	–	–	0.71	–
Other appliances	–	–	–	0.38	–
Cleanliness of area where food is stored	–	–	–	0.49	–
Garbage disposal	–	–	–	0.36	–
Condition of area for excreta	–	–	–	0.25	–
Lighting	–	–	–	0.91	–
Housing material	–	–	–	0.66	–
Neighborhood excreta removal	–	–	–	0.56	–
Neighborhood garbage removal	–	–	–	0.61	–
Beds	–	–	–	0.70	–
Boat	–	–	–	−0.02	–
Cattle (cows or carabaos)	–	–	–	−0.32	–
Farm animals (goat, horse, pigs etc)	–	–	–	−0.31	–
Other vehicles (banca, motorcycle or tricycle etc)	–	–	–	0.18	–
Truck or bus	–	–	–	0.52	–
Microwave	–	–	–	–	0.76

Harmonized asset indices were created separately for each site.

**Fig. 1:**
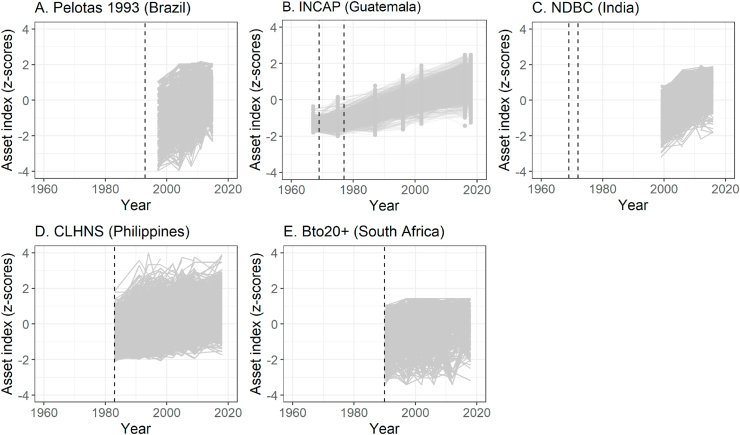
Household-level trends in temporally-harmonized asset index for birth cohorts from low- and middle-income countries.

**Fig. 2:**
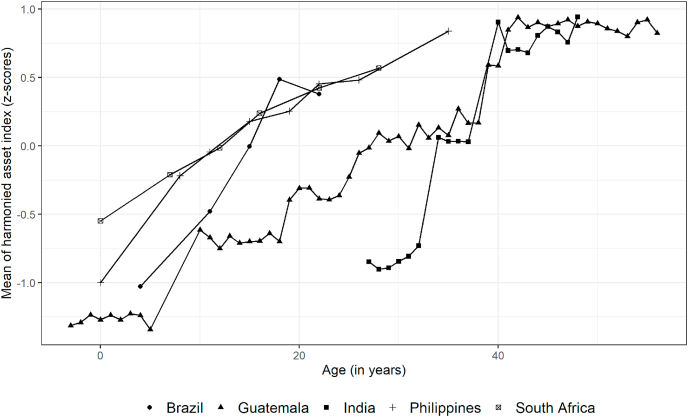
Mean trends in temporally-harmonized asset index for birth cohorts All values are mean values from a harmonized index created separately for each cohort. Only mean values at ages where number of observations are greater than 30 are plotted. Guatemala has age ranges of 0–5 in 1967 and 0–7 in 1975 which have been combined (ages less than zero indicate those born after data collection). The number of data points above for Guatemala (1962–1977) and India (1969–1972) is a result of the wide range of birth years. Missing birth years were imputed with median of data collection (i.e. 1971) in India.

The temporally-harmonized asset index suggested that wealth improved over time on average (Table 3) for all birth cohorts (Brazil: 1.03 to 0.38; Guatemala: 1.31 to 0.91; India: 0.86 to 0.84; Philippines: 1.00 to 0.84; South Africa: 0.55 to 0.57). Though most households improved their living standards over time, there was heterogeneity in asset accumulation (Fig. 1). Wealth heterogeneity between households (as measured by sample standard deviation; SD) at each wave was relatively stable between birth and adolescence except in Philippines (0.64 at 0y to 0.91 at 12-13y) and South Africa (1.00 at 0-2y to 0.85 at 12-13y). From adolescence to adulthood, wealth heterogeneity (SD) declined in all cohorts except Guatemala (0.42 at 10-25y to 0.66 at 40-57y). Wealth also increased for all cohorts as the participants grew older (Fig. 2).

**Table 3:** Summary of harmonized index over time for COHORTS.

	Pelotas 1993 (Brazil)		INCAP (Guatemala)		NDBC (India)		CLHNS (Philippines)		Birth to Twenty plus (South Africa)	
	Age at wave	Summary	Age at wave	Summary	Age at wave	Summary	Age at wave	Summary	Age at wave	Summary
Mean ± SD										
1	3–4	−1.03 ± 1.15	0–5	−1.31 ± 0.28	27–33	−0.86 ± 0.65	0	−1.00 ± 0.64	0–2	−0.55 ± 1.00
2	11–12	−0.48 ± 1.06	0–7	−1.15 ± 0.34	34–40	0.03 ± 0.79	7–8	−0.20 ± 0.92	7–8	−0.21 ± 0.90
3	13–14	−0.00 ± 0.88	10–25	−0.69 ± 0.42	40–47	0.72 ± 0.75	12–13	−0.05 ± 0.91	12–13	−0.01 ± 0.85
4	18	0.49 ± 0.75	19–34	−0.12 ± 0.47	44–51	0.84 ± 0.67	15–16	0.18 ± 0.90	16–17	0.24 ± 0.88
5	22	0.38 ± 0.66	25–40	0.15 ± 0.53			18–19	0.25 ± 0.80	22–23	0.42 ± 0.91
6			37–55	0.85 ± 0.68			21–22	0.45 ± 0.84	27–28	0.57 ± 0.80
7			40–57	0.91 ± 0.66			25–26	0.48 ± 0.84		
8							33–36	0.84 ± 0.86		
Median [IQR]										
1	3–4	−0.85 [-1.26, −0.43]	0–5	−1.31 [-1.53, −1.08]	27–33	−0.88 [-1.19, −0.34]	0	−1.15 [-1.48, −0.67]	0–2	−0.47 [-1.22, 0.20]
2	11–12	−0.67 [-1.16, 0.08]	0–7	−1.10 [-1.38, −0.96]	34–40	0.12 [-0.59, 0.63]	7–8	−0.27 [-0.96, 0.43]	7–8	−0.15 [-0.74, 0.42]
3	13–14	0.01 [-0.70, 0.52]	10–25	−0.67 [-0.94, −0.43]	40–47	0.85 [0.23, 1.32]	12–13	−0.04 [-0.70, 0.63]	12–13	0.03 [-0.52, 0.57]
4	18	0.54 [0.09, 0.98]	19–34	−0.14 [-0.42, 0.20]	44–51	0.98 [0.47, 1.34]	15–16	0.24 [-0.47, 0.83]	16–17	0.34 [-0.33, 0.98]
5	22	0.44 [0.00, 0.85]	25–40	0.09 [-0.20, 0.50]			18–19	0.28 [-0.30, 0.80]	22–23	0.61 [0.03.1.06]
6			37–55	0.83 [0.37, 1.35]			21–22	0.46 [-0.15, 0.98]	27–28	0.83 [0.12, 1.20]
7			40–57	0.92 [0.48, 1.38]			25–26	0.46 [-0.06, 0.97]		
8							33–36	0.77 [0.27, 1.37]

Visual inspection of the histograms of harmonized wealth (Supplementary Figs. 2A–E) at different study waves suggest clumping for Brazil (12 assets) and South Africa (10 assets). We also observed some left-truncation in Guatemala for 1967 (age 0-5y) and 1975 (age 0-7y) suggesting a failure to differentiate among the poorest households.

### 3.2 Validation of harmonized index

Our validation exercise suggested that cross-sectional asset indices restricted to the set of common assets used to construct the temporally-harmonized index were correlated with the harmonized index (Table 4). All correlations were greater than 0.90 except for Brazil in 2015 (*r=*0.83) and India in 1999–00 (r = 0.85) and 2016–19 (r = 0.82). Comparison of asset loadings of harmonized index to cross-sectional asset indices created using same set of assets suggest that loadings were the same for Philippines and South Africa, with moderate or high congruence (phi >0.85) for most waves across other sites except India (Supplementary Tables 3A–E, Supplementary Table 4).

**Table 4:** Correlation of harmonized index with cross-sectional indices created from same set of assets for COHORTS.

		Pelotas 1993 (Brazil)		INCAP (Guatemala)		NDBC (India)		CLHNS (Philippines)		Birth to Twenty plus (South Africa)
	Age at wave	rho	Age at wave	rho	Age at wave	rho	Age at wave	rho	Age at wave	rho
1	3–4	0.96	0–5	0.95	27–33	0.85	0	0.99	0–2	0.99
2	11–12	0.97	0–7	0.91	34–40	0.93	7–8	1.00	7–8	0.99
3	13–14	0.98	10–25	0.92	40–47	0.91	12–13	0.99	12–13	0.99
4	18	0.99	19–34	0.96	44–51	0.82	15–16	0.99	16–17	0.99
5	22	0.83	25–40	0.98			18–19	0.99	22–23	0.95
6			37–55	0.99			21–22	0.99	27–28	0.98
7			40–57	0.98			25–26	0.99		
8							33–36	0.99		

All values are Spearman rank correlations. Correlation of harmonized index with cross-sectional indices created from all available assets is available in Supplementary Tables 3A–E.

Asset loadings varied over time, with some becoming common (like electricity or television in Philippines), others becoming rare (like radio in Guatemala) or being substituted with novel assets (like coolers with air conditioners in India). For example, in the cross-sectional index for Brazil in 2015, single-door refrigerator loadings were negative (−0.24 vs 0.54 in harmonized index) since households which possessed a duplex refrigerator (0.65 vs 0.67 in harmonized index) were less likely to possess a single-door refrigerator relative to previous waves. Cross-sectional indices created using all available assets for each study wave (Supplementary Tables 3A–E) were also correlated with the harmonized index for all cohorts (Brazil: r = 0.78 to 0.96; Guatemala: r = 0.81 to 0.95; India: 0.75 to 0.93; Philippines: r = 0.92 to 0.99; South Africa: r = 0.84 to 0.96). The lower correlation of the harmonized index with cross-sectional indices could be due to three reasons: newer assets (such as employing a cleaner and clothes dryer in Brazil, or plasma TV and internet in India or microwave in South Africa), removing low-variance assets (such as car, motorcycle and sewage facility in Guatemala), or assets not being collected in some waves (such as radio, toilet and water source in South Africa in 2012–13). Cross-sectional indices (Supplementary Tables 5A–C) created for urban and rural strata were correlated with the temporally-harmonized index for Philippines (Rural: r ≥ 0.95; Urban: r ≥ 0.98) and Guatemala (r ≥ 0.95).

Maternal schooling was correlated with harmonized asset index in childhood (r = 0.15 to 0.56) and school-age (r = 0.28 to 0.57) in for all cohorts (Table 5). Attained schooling was correlated (r = 0.18 to 0.54) with harmonized index in late-adolescence and early adulthood (15–40y) for all cohorts. Attained schooling was also correlated with harmonized index in middle adulthood for Guatemala (r = 0.45) and India (r = 0.40 to 0.44). Correlations of harmonized wealth in childhood with height-for-age z-scores at 24 months (r = 0.11 to 0.27) were small but positive in three cohorts. Similarly, harmonized wealth in adulthood were correlated with adult body mass index (r = 0.15 to 0.21) in the older three (out of 5) cohorts. These findings, similar to that of cross-sectional wealth (Supplementary Table 6), suggest construct validity of the harmonized wealth measure.

**Table 5:** Correlation of schooling and health measures with harmonized asset index in corresponding wave among those who participated in adulthood.

		Pelotas 1993 (Brazil)		INCAP (Guatemala)		NDBC (India)		CLHNS (Philippines)		Birth to Twenty plus (South Africa)
	Age at wave	rho	Age at wave	rho	Age at wave	rho	Age at wave	rho	Age at wave	rho
Schooling										
1	3–4^a^	0.54	0–7^a^	0.15	0–2^b^	Not available	0^a^	0.56	0–2^a^	0.23
2	11–12^a^	0.57	10–25	0.31	27–33	0.44	7–8^a^	0.56	7–8^a^	0.28
3	13–14^a^	0.56	19–34	0.31	34–40	0.46	12–13^a^	0.54	12–13^a^	0.29
4	18	0.45	25–40	0.36	40–47	0.40	15–16	0.50	16–17	0.18
5	22	0.42	37–55	0.45	44–51	0.44	18–19	0.52	22–23	0.18
6			40–57	0.45			21–22	0.52	27–28	0.23
7							25–26	0.54		
8							33–36	0.53		
HAZ at 2y										
9	2^b^	Not available	2	0.11	2^b^	Not available	2	0.27	2	0.13
BMI in adulthood										
10	22	−0.05	37–55	0.15	44–51	0.21	33–36	0.19	22–23	0.06

Sample sizes among those who participated in adulthood varied for above Pearson correlations: Brazil (995; 3608; 3576; 3519; 3805; 3559), Guatemala (1346; 931; 641; 821; 1160; 1265; 723; 1143), India (868; 807; 790; 841; 828), Philippines (1326; 1321; 1325; 1325; 1303; 1311; 1274; 1249; 1326; 1285; 1304), and South Africa (1132; 999; 1071; 1201; 1274; 1393; 856; 1202). This is not the sample size of participants at each wave (non-monotone missingness).

a Correlation with maternal schooling. Values from 1967 to 1975 were combined for Guatemala (n = 2392).

b Temporally harmonized asset index was not available in childhood for NDBC and before 3 years for Pelotas 1993

### 3.3 Sensitivity analysis

The benchmark asset index was robust to pairwise dropping of assets (Brazil: r ≥ 0.95; Guatemala: r ≥ 0.99; India: r ≥ 0.85; Philippines: r ≥ 0.99; South Africa: r ≥ 0.91) as well as survey years (Brazil: r ≥ 0.99; Guatemala: r = 1.00; India: r ≥ 0.88; Philippines: r = 1.00; South Africa: r ≥ 0.99). The index was also robust to joint dropping of asset and survey year (Brazil: r ≥ 0.96; Guatemala: r ≥ 0.99; India: r ≥ 0.85; Philippines: r ≥ 0.99; South Africa: r ≥ 0.97). Additional information is available in Supplementary File 1.

Finally, the benchmark asset index was invariant to alternate factor extraction procedures (Supplementary Table 7). Asset indices created using Exploratory Factor Analysis with polychoric (r ≥ 0.94) or Pearson's correlation (r ≥ 0.94) matrix, Principal Components Analysis using Pearson's correlation matrix (r ≥ 0.99) or Multiple Correspondence Analysis (r ≥ 0.98) were rank correlated with the benchmark index for all countries.

## 4 Discussion

Our results suggest that a harmonized index, created using consistently collected measures of asset ownership and housing characteristics, may be used to study trajectories of household wealth mobility within birth cohorts from LMIC settings. Such a temporally-harmonized asset index could then be used to study the association of wealth gains at different stages of the life course with health and wellbeing outcomes in later life (Duc, 2019). Across all cohorts, households acquired additional assets and improved their housing characteristics over time. Previous research from our team used the INCAP cohort (Guatemala) to develop the approach for temporally harmonized index construction and validation (Varghese et al., 2021). Our results from this analysis complements previous research by generalizing findings that temporally harmonized asset indices, created from a shorter set of assets for cohort studies, are rank-correlated with the standard approach of creating asset-based indices across different geographical contexts (Duc, 2019; Harttgen et al., 2013; Östberg et al., 2018). The temporally harmonized asset index, created for cohort studies, using consistently collected set of assets, complements previous research that studied how mean household wealth improved over time across different countries using cross-sectional nationally-representative surveys (Hruschka et al., 2015; Rutstein & Staveteig, 2013; Smits & Steendijk, 2014).

Our results also suggest that an index created from a subset of these assets was correlated with the cross-sectional asset indices (created using all available assets) used in epidemiological studies as a proxy for wealth and standard of living. The harmonized index also correlated with cross-sectional indices created separately for urban and rural samples in Philippines and Guatemala. The mean values of harmonized index in urban areas were higher than rural areas for all study years in Guatemala and Philippines (results not shown). The harmonized index also displayed construct validity when compared with maternal schooling and attained schooling in early life and adulthood respectively.

We observed clumping in Brazil and South Africa due to unavailability of consistently collected assets that could adequately differentiate households. We observed left-truncation in the earlier study waves (in 1967 and 1975) from Guatemala potentially due to unavailability of assets that are able to differentiate between poor households. One reason for this is that our cohort originally belonged to rural villages that were predominantly reliant on agriculture, and gradually transitioned to manufacturing and service sector jobs over time (Hackman et al., 2020; Melgar et al., 2020). Asset-based indices are known to be biased against households that derive livelihoods from the agricultural economy. Households within these villages being uniformly poor at the beginning of the study could be another reason for the observed distribution (Melgar et al., 2020).

The harmonized index was correlated with indices derived from dropping pairs of assets or survey years as well as combinations of asset and survey year, consistent with the International Wealth Index and results from the Millennium Villages Project (Michelson et al., 2013; Smits & Steendijk, 2014). Also consistent with other studies, an index extracted using PCA of the polychoric correlation matrix was highly correlated with indices extracted using other approaches (Exploratory Factor Analysis, Multiple Correspondence Analysis) (Amek et al., 2015; Chasekwa et al., 2018; Howe et al., 2008; Michelson et al., 2013). Moreover, assets related to livestock, i.e., poultry, cattle and farm animals, had negative loadings on the harmonized index as well as cross-sectional indices for Philippines (but loaded on other components), similar to research from South Africa and Kenya (Balen et al., 2010; Chuma & Molyneux, 2009; Vollmer & Alkire, 2020; Wittenberg & Leibbrandt, 2017). Dropping these assets did not change our results. However, our index may fail to capture non-engagement with the modern cash-oriented sectors (but engaged with the agricultural sector) by some cohort members who possessed substantial livestock wealth (Bingenheimer, 2007).

### 4.1 Limitations

The index has limitations inherent to the longitudinal nature of our study. The harmonized asset index assumes that the structure of interrelationships among different assets is similar over time. However, the same asset changes in importance over time as it becomes ubiquitous or less common. Our analysis of congruence suggests that the harmonized index is similar to the cross-sectional index for most study waves. Though all birth cohorts experienced significant attrition over the life course, comparison of early life characteristics of cohort members who did not participate suggested that they were otherwise similar. Additionally, since the index is relative and country-specific, it does not explain the association of absolute wealth gains (such as savings or debt) across the life course with health outcomes. Such studies have been attempted in a limited way across geographies such as in the analysis of cross-sectional measures of income (such as gross national income per capita adjusted for purchasing power parity) or household wealth with child height and adult overweight (Karra et al., 2017; Lartey et al., 2019; Paciorek et al., 2013; Templin et al., 2019). The positive association between wealth and BMI in adulthood in Guatemala, India and Philippines is consistent with wider literature that suggests countries earlier in the nutrition transition exhibit a positive association between socioeconomic position and BMI (Templin et al., 2019).

Since our analysis was restricted to assets collected over the life course, we could not include newer electronic goods such as digital tablets and laptops. Data on assets were not available in early life for India. The limited availability of asset data also prevented us from inferring if other metrics associated with assets – quantity, quality or functioning, technological generation, availability of substitutes – biased our findings (Johnston & Abreu, 2016; Merola & Baulch, 2018). Our sensitivity analysis using data from the Pelotas 1993 (Brazil) cohort suggested that a harmonized asset index created using counts of assets such as televisions, cars and housekeepers as well as number of bathrooms in the house was correlated (r = 0.98) with the benchmark asset index. Similar to cross-sectional surveys, we assume that all assets are public goods, i.e. available to all members in the household and except for number of rooms per member, do not adjust for household size and composition (Poirier et al., 2019). We do not account for selection of individuals into households with higher/lower asset index (e.g. from rural to urban areas for employment) and changing households (e.g. for marriage) that could result in scores that are different from what would be concurrently experienced by their original family unit (household of birth).

## 5 Conclusion

Temporally-harmonized asset indices open up opportunities for longitudinal investigation of the impact of early life wealth on later life health outcomes. Such indices allow comparison of wealth at different life stages in the same measurement unit under assumptions of temporal validity. Previous studies exploring the link between economic and epidemiological transition rely on measures of material well-being which are ecological such as Gross National Income per capita (Jaacks et al., 2019) or cross-sectional wealth (Templin et al., 2019). However, household wealth (both relative and absolute) at different stages of life course may determine behaviors such as physical activity or diet or psychosocial resources such as self-efficacy and life satisfaction that are associated with health (Lynch et al., 2000; Marmot & Wilkinson, 2001). Exploring the association of household wealth with health at different stages of the life course could also aid in designing social safety nets targeting specific health outcomes. Moreover, studies in LMICs exploring the roles of these downstream pathways (such as health behaviors and psychosocial stressors) may be confounded by life course wealth (and other measures of SEP) which ought to be quantified.

Consistently administered and contextually relevant measures of wealth may inform design of interventions and better estimation of long-term effects of life course exposures on health and human capital in low- and middle-income countries.

## Author contributions

JSV, ADS: conceptualized the study; JSV, LSA, SAP and ADS: developed the methodology and led the writing team; JSV: performed the statistical analysis and wrote the first draft; LSA, SAB, SKB, DBC, BLH, NPL, RM, AMBM, SAN, LMR, MRZ, HSS, FCW, ADS: led and participated in data collection; all authors: read and commented on successive drafts; and all authors: read and approved the final manuscript.

## Data availability statement

The code for the analysis is available on https://github.com/jvargh7/cohorts-asset-harmonization. Data will be available upon reasonable request addressed to the principal investigators at each study site.

## Ethics approval and consent to participate

All participants provided written informed consent prior to participation at each study wave. Ethical approval for the latest study wave for the cohorts were obtained from the Federal University of Pelotas, Brazil (Protocol 1.250.366), Institutional Review Board of Emory University, USA (Protocol 95960), Institute of Nutrition for Central America and Panama, Guatemala (Protocol CIE-REV-072-2017), Sitaram Bhartia Institute of Science and Research, India (SBISR/2012/002; SBISR/RES1/3/2012; SBISR/IEC/2014/001; IEC/SBISR/2015/1; FL/SBISR/IEC/2019–01), Research Ethics Committee at University of San Carlos, Philippines (Protocol 006/2018-01-borja), and Human Research Ethics Committee at University of Witswatersrand, South Africa (Certificate No. M180225).

## Consent for publication

Not applicable.

## Funding

Funded by the Bill and Melinda Gates Foundation [grant number OPP1164115] for data collection in Guatemala, Philippines, and South Africa and India for data management and analysis. Data collection in Brazil was funded by the 10.13039/501100009053Wellcome Trust [grant number 086974/Z/08/Z]. The New Delhi Birth Cohort has received funding from the 10.13039/501100001411Indian Council of Medical Research [grant numbers 50/1-3/TF/05-NCD-II, 3/1/2/2/15-RCH, 5/10/FR/10/2019-RCH, 5/4/8-7/2019-NCD-II], the 10.13039/501100001407Department of Biotechnology [grant numbers BT/PR3874/MED/97/1/2011, BT/PR5317/FNS/20/552/2012], the United States National Center for Health Statistics [grant numbers PL-480, RESEARCH PROJECT 0-1-658-2], and the British Heart Foundation [grant number UKPG/05/046]. The Birth to Twenty Plus Cohort is supported by the South African Medical Research Council, DSI-NRF Centre of Excellence in Human Development at the University of the Witwatersrand, Johannesburg, South Africa, and the 10.13039/501100009053Wellcome Trust [grant numbers 077210/Z/05/Z, 092097/Z/10/Z].

## Declaration of competing interest

None declared.
