# Supplementary material for: Changes in asset-based wealth across the life course in birth cohorts from five low- and middle-income countries
Source: SSM Popul Health. 2021 Nov 20;16:100976. doi: 10.1016/j.ssmph.2021.100976 (PMC8637637; doi:10.1016/j.ssmph.2021.100976)
Supplement: Multimedia component 2 [file mmc2.docx]

Contents

[Supplementary Fig 1. Trajectories of gross-domestic product (GDP) per capita and Gini index for five low- and middle-income countries 3](#_Toc79662543)

[Supplementary Fig 2A. Distribution of harmonized wealth index over time in Pelotas 1993 cohort by study wave 4](#_Toc79662544)

[Supplementary Fig 2B. Distribution of harmonized wealth index over time in INCAP Longitudinal Study cohort by study wave 5](#_Toc79662545)

[Supplementary Fig 2C. Distribution of harmonized wealth index over time in New Delhi Birth cohort by study wave 6](#_Toc79662546)

[Supplementary Fig 2D. Distribution of harmonized wealth index over time in Cebu Longitudinal Health and Nutrition Study by study wave 7](#_Toc79662547)

[Supplementary Fig 2E. Distribution of harmonized wealth index over time in Birth to Twenty plus cohort by study wave 8](#_Toc79662548)

[Supplementary Note 1. Detailed statistical methods 9](#_Toc79662549)

[Supplementary Table 1A. Categorization and availability of assets for Pelotas 1993 cohort by study wave 11](#_Toc79662550)

[Supplementary Table 1B. Categorization and availability of assets for INCAP Longitudinal Study cohort by study wave 13](#_Toc79662551)

[Supplementary Table 1C. Categorization and availability of assets for New Delhi Birth Cohort by study wave 16](#_Toc79662552)

[Supplementary Table 1D. Categorization and availability of assets for Cebu Longitudinal Health and Nutrition Study by study wave 19](#_Toc79662553)

[Supplementary Table 1E. Categorization and availability of assets for Birth to Twenty plus cohort by study wave 26](#_Toc79662554)

[Supplementary Table 2A. Comparison of early life characteristics for Pelotas 1993 cohort for non-participants in study wave 28](#_Toc79662555)

[Supplementary Table 2B. Comparison of early life characteristics for INCAP Longitudinal Study cohort for participants in study wave 30](#_Toc79662556)

[Supplementary Table 2C. Comparison of early life characteristics for New Delhi Birth Cohort for participants in study wave 31](#_Toc79662557)

[Supplementary Table 2D. Comparison of early life characteristics for Cebu Longitudinal Health and Nutrition Study for participants in study waves 32](#_Toc79662558)

[Supplementary Table 2E. Comparison of early life characteristics for Birth to Twenty plus cohort for participants in study waves 33](#_Toc79662559)

[Supplementary Table 3A. Loadings of harmonized index and cross-sectional indices with all assets for Pelotas 1993 cohort 34](#_Toc79662560)

[Supplementary Table 3B. Loadings for harmonized index and cross-sectional indices with all assets for INCAP Longitudinal Study 36](#_Toc79662561)

[Supplementary Table 3C. Loadings for harmonized index and cross-sectional indices with all assets for New Delhi Birth Cohort 38](#_Toc79662562)

[Supplementary Table 3D. Loadings for harmonized index and cross-sectional indices with all assets for Cebu Longitudinal Health and Nutrition Study 40](#_Toc79662563)

[Supplementary Table 3E. Loadings for harmonized index and cross-sectional indices with all items for Birth to Twenty plus cohort 44](#_Toc79662564)

[Supplementary Table 4. Tucker index of congruence between harmonized index and cross-sectional asset indices created using same set of covariates 46](#_Toc79662565)

[Supplementary Table 5A. Loadings for harmonized index and cross-sectional indices with same assets as harmonized index for INCAP Longitudinal Study by Urban and Rural strata 47](#_Toc79662566)

[Supplementary Table 5B. Loadings for harmonized index and cross-sectional indices with same assets as harmonized index for Cebu Longitudinal Health and Nutrition Study for Rural strata 49](#_Toc79662567)

[Supplementary Table 5C. Loadings for harmonized index and cross-sectional indices with same assets as harmonized index for Cebu Longitudinal Health and Nutrition Study for Urban strata 51](#_Toc79662568)

[Supplementary Table 6. Correlation of schooling and health measures with cross-sectional asset index in corresponding wave among those who participated in adulthood 53](#_Toc79662569)

[Supplementary Table 7. Correlation of harmonized index with alternate factor extraction procedures 54](#_Toc79662570)

# Supplementary Fig 1. Trajectories of gross-domestic product (GDP) per capita and Gini index for five low- and middle-income countries


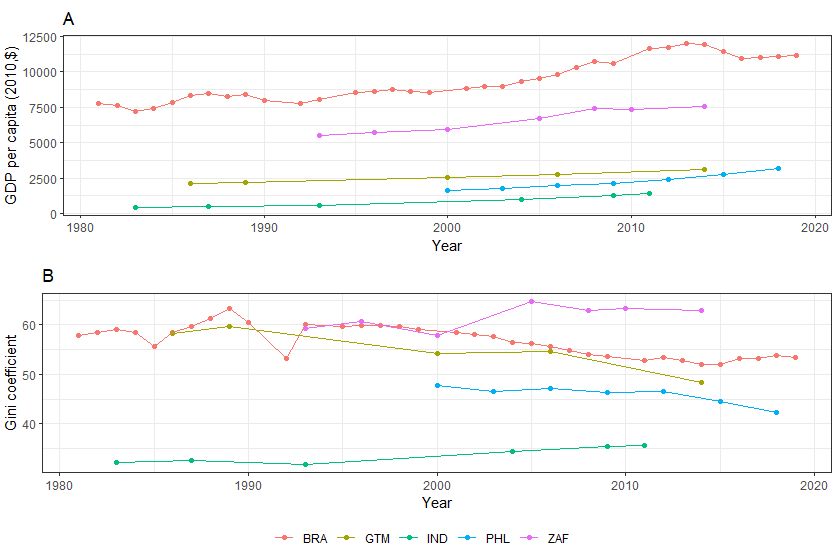


Data from World Bank World Development Indicators

# Supplementary Fig 2A. Distribution of harmonized wealth index over time in Pelotas 1993 cohort by study wave


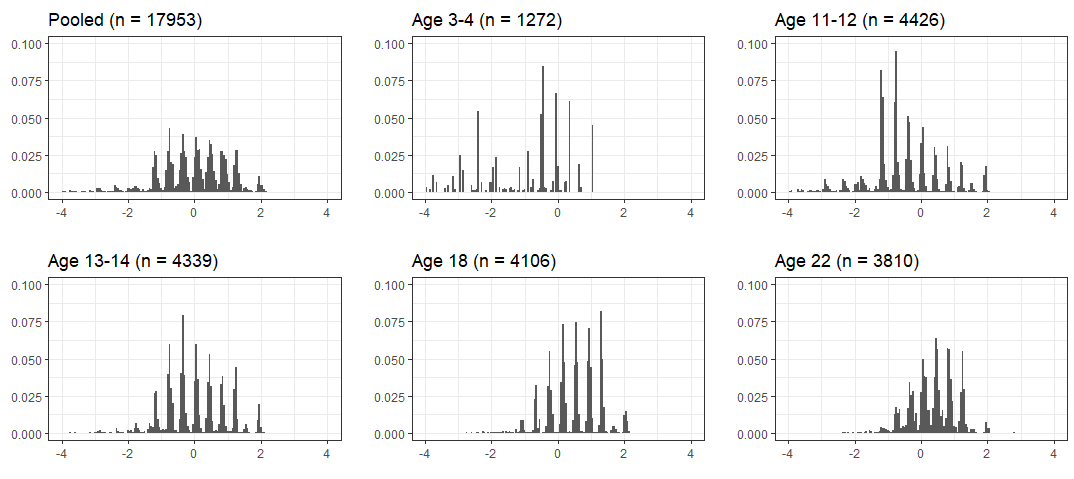


# Supplementary Fig 2B. Distribution of harmonized wealth index over time in INCAP Longitudinal Study cohort by study wave


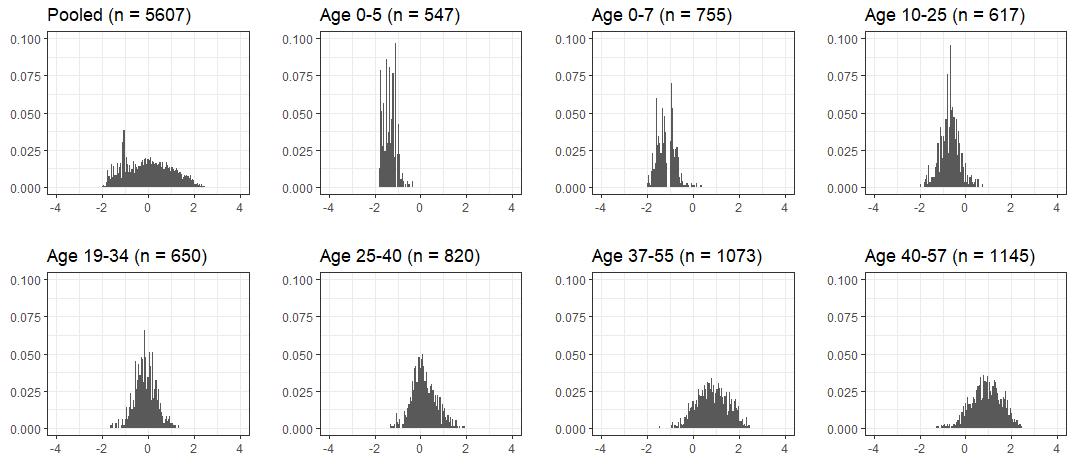


# Supplementary Fig 2C. Distribution of harmonized wealth index over time in New Delhi Birth cohort by study wave


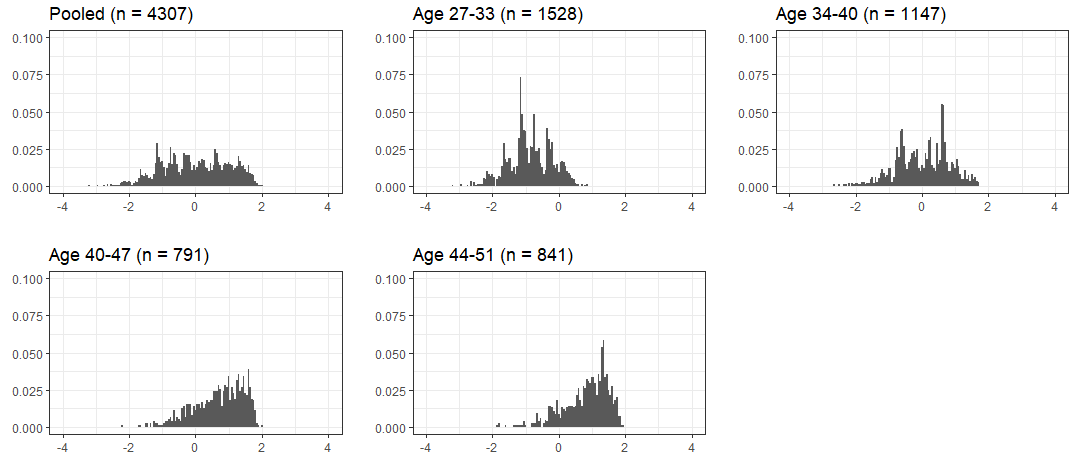


# Supplementary Fig 2D. Distribution of harmonized wealth index over time in Cebu Longitudinal Health and Nutrition Study by study wave


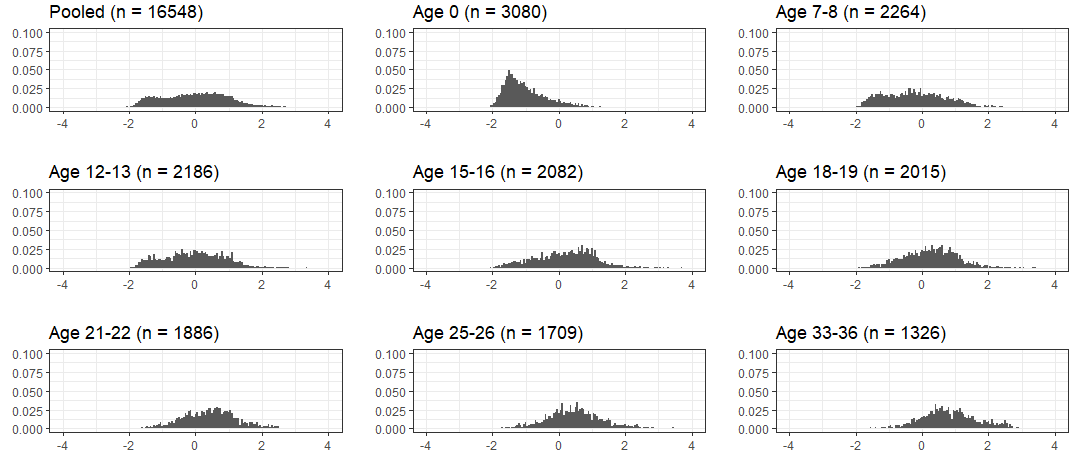


# Supplementary Fig 2E. Distribution of harmonized wealth index over time in Birth to Twenty plus cohort by study wave


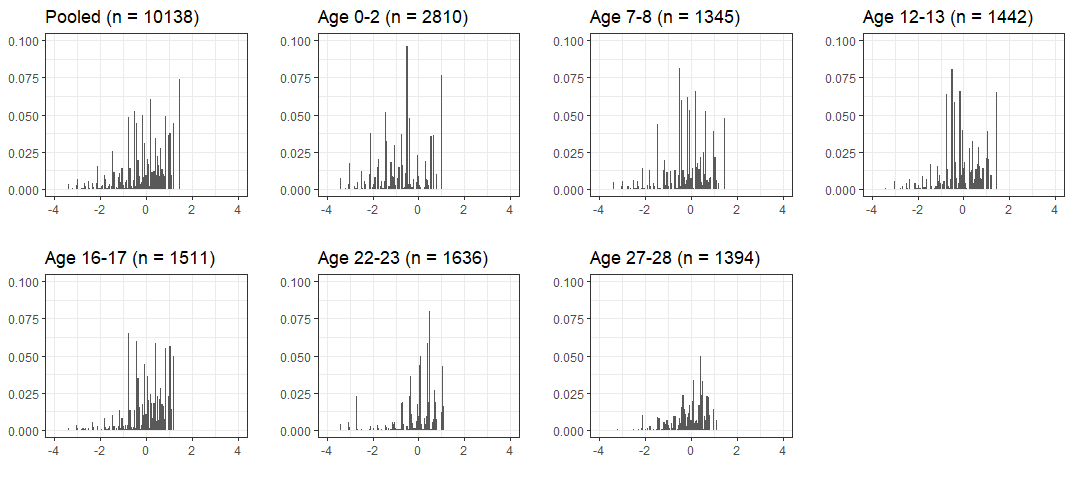


# Supplementary Note 1. Detailed statistical methods

**A. Description of statistical procedure used in estimation of temporally harmonized index**

We followed the following steps to construct the harmonized index across study waves (henceforth ‘waves’) over the life course. Individuals were the unit of analysis (except for Guatemala where we used households), and analyses were restricted to individuals who participated in the study wave. Our analysis for the harmonized index was on a pooled dataset across all waves separately for each cohort.

1. All assets and housing characteristics (henceforth ‘items’) available for each cohort was harmonized by categorizing them into ownership (yes/no; binary), quality (low/medium/high; ordinal) or quantity (counts; integer/continuous) variables.

2. We then identified those items that were available in all waves, or missing in at most one wave. These items were considered for further analysis.

3. Imputation: We imputed the items that were missing in an entire wave with the preceding wave for those individuals who participated in both waves. If an individual did not participate in the preceding wave, we imputed the value with the cross-sectional mode. If an item was missing in the first wave, we imputed it with zero. We assessed robustness of the index at a later stage by dropping two assets and study years at a time.

4. We constructed a mixed correlation matrix (Pearson for continuous, polychoric for ordinal, tetrachoric for binary, polyserial/biserial correlations for ordinal/binary with continuous) for the imputed dataset.

5. We used principal component analysis (PCA) and extracted the first component as the harmonized index. We standardized the index (to unit variance) the component by subtracting by the pooled mean and dividing by standard deviation.

**B.** **Description of statistical procedure used for sensitivity analysis for cross-sectional indices**

1. We identified all assets that were available in a cross-sectional wave as part of the sensitivity analysis comparing the harmonized index with the normative cross-sectional indices.

2. We imputed the value of an item with the cross-sectional mode for those who did not provide information in the cross-sectional wave or the preceding wave for cross-sectional indices similar to the harmonized index. For the normative cross-sectional indices that include all assets collected in a wave, we included only those assets which were collected in the wave and imputed missing values with the mode.

3. We removed those variables which displayed near-zero variance (ratio of most common to second most common category > 95:5). We limited our analysis to variables that displayed sufficient variance in the data. This was different from the harmonized index wherein we included all variables irrespective of their variance.

4. We constructed a mixed correlation matrix (Pearson for continuous, polychoric for ordinal, tetrachoric for binary, polyserial/biserial correlations for ordinal/binary with continuous) for the imputed dataset.

5. We used principal component analysis (PCA) and extracted the first component as the harmonized index. We standardized the index (to unit variance) the component by subtracting by the pooled mean and dividing by standard deviation.

# Supplementary Table 1A. Categorization and availability of assets for Pelotas 1993 cohort by study wave

|  | **Survey Year** | **1997** | **2004** | **2008** | **2011** | **2015** |  |  |  |
| --- | --- | --- | --- | --- | --- | --- | --- | --- | --- |
|  | ***Percentage of original sample with asset data*** | 24.2% | 84.3% | 82.7% | 78.2% | 72.6% |  |  |  |
| **Asset** | **Categorization** |  |  |  |  |  |  |  |  |
| Car | Yes, No | 30.7 | 35.3 | 37.7 | 46.2 | 57.9 |  |  |  |
| Computer | Yes, No |  | 16.9 | 45.1 | 76.7 | 76.8 |  |  |  |
| Duplex refrigerator | Yes, No |  | 28.9 | 35.6 | 44.2 | 52.2 |  |  |  |
| DVD player | Yes, No | 32.5 | 37.2 | 81.2 | 92.9 | 78.5 |  |  |  |
| Housekeeper | Yes, No | 9.3 | 6.3 | 5.4 | 5.5 | 2.8 |  |  |  |
| Radio | Yes, No | 91.6 | 89.8 | 90.3 |  | 74.1 |  |  |  |
| Refrigerator | Yes, No | 87.8 | 91.7 | 94.9 | 99.1 | 92.8 |  |  |  |
| Television | Yes, No | 82.4 | 93.3 | 98.3 | 99.2 | 99.5 |  |  |  |
| Vacuum cleaner | Yes, No | 18.6 | 22.7 | 27.0 | 44.3 |  |  |  |  |
| Washing machine | Yes, No | 46.5 | 60.2 | 64.5 | 75.3 | 80.8 |  |  |  |
| Piped water | Low: Not available, Other location | 3.5 | 1.1 | 0.8 |  | 0.3 |  |  |  |
|  | Medium: In courtyard, artesian well/spring | 4.6 | 2.2 | 0.8 |  | 1.7 |  |  |  |
|  | High: Inside home, general distribution network | 91.8 | 96.7 | 98.4 |  | 98.0 |  |  |  |
| House ownership | Yes, No |  | 81.4 | 83.2 | 86 |  |  |  |  |
| Housing material | Low: Cardboard/tin, clay, canvas, Other | 0.1 | 4.1 |  |  |  |  |  |  |
|  | Medium: Wood (regular/irregular), mixed, brick without plaster | 20.9 | 19.8 |  |  |  |  |  |  |
|  | High: Brick with plaster, apartment | 79.0 | 76.1 |  |  |  |  |  |  |
| Toilet | Low: None | 8.3 | 2.1 | 0.9 |  |  |  |  |  |
|  | Medium: Toilet without flush, Outside house/cesspool | 7.5 | 2.6 | 1.4 |  |  |  |  |  |
|  | High: Flush toilet | 84.1 | 95.3 | 97.6 |  |  |  |  |  |
| Air conditioning | Yes, No |  |  |  | 15.5 | 32.5 |  |  |  |
| Cleaning lady | Yes, No |  |  |  |  | 6.2 |  |  |  |
| Clothes dryer | Yes, No |  |  |  |  | 22.8 |  |  |  |
| Desktop | Yes, No |  |  |  | 65.1 |  |  |  |  |
| Dishwasher | Yes, No |  |  |  |  | 4.7 |  |  |  |
| Internet | Yes, No |  | 76.7 | 71.3 | 82.4 | 85.2 |  |  |  |
| Microwave | Yes, No |  |  |  | 62.5 | 78.1 |  |  |  |
| Motorcycle | Yes, No | 8.9 |  |  |  | 34.1 |  |  |  |
| Notebook computer | Yes, No |  |  |  | 38.8 |  |  |  |  |
| Stereo | Yes, No |  | 64.9 | 63 |  |  |  |  |  |
| Street is paved | Yes, No |  |  |  |  | 50.5 |  |  |  |
| Video game | Yes, No |  |  | 33.6 | 45.4 |  |  |  |  |

# Supplementary Table 1B. Categorization and availability of assets for INCAP Longitudinal Study cohort by study wave

|  | **Survey Year** | **1967** | **1975** | **1987** | **1996** | **2002** | **2015-16** | **2017-18** |  |
| --- | --- | --- | --- | --- | --- | --- | --- | --- | --- |
|  | ***Percentage of original sample with asset data*** | 67.0% | 92.5% | 56.9% | 35.7% | 44.0% | 48.6% | 52.9% |  |
| **Asset** | **Categorization** |  |  |  |  |  |  |  |  |
| Bicycle | Yes, No | 0.7 | 2.5 | 10.9 | 44.6 | 53.2 | 50.9 | 45.2 |  |
| Car | Yes, No |  | 0.1 | 0.8 | 3.1 | 7.7 | 27 | 26.7 |  |
| Electricity | Yes, No | 0.2 | 10.9 | 71.6 | 92 | 95.6 | 97.9 | 97.5 |  |
| Motorcycle | Yes, No |  | 0.3 | 0.8 | 2 | 1.2 | 23.7 | 30.7 |  |
| House ownership | Yes, No | 84.1 | 80 | 82.2 | 75.4 | 78.2 | 81.3 | 83.1 |  |
| Radio | Yes, No | 32.2 | 52.8 | 58 | 72.8 | 21.1 | 30.2 | 21.6 |  |
| Refrigerator | Yes, No | 0.5 | 2.4 | 3.9 | 15.1 | 27.2 | 67 | 71.3 |  |
| Sewing machine | Yes, No |  | 10.7 | 10.2 | 10.8 | 10.4 | 19.2 | 18.2 |  |
| Television | Yes, No |  | 0.9 | 22.4 | 65.8 | 77.1 | 92 | 92.3 |  |
| Floor quality | Low: Earth | 96 | 88.6 | 62.9 | 37.2 | 23.7 | 9.9 | 9.2 |  |
|  | Medium: Brick or clay, cement cake | 4 | 8.9 | 32.7 | 53.1 | 59.9 | 55.2 | 57 |  |
|  | High: Mosaic, wood |  | 2.5 | 4.4 | 9.7 | 16.5 | 35 | 33.8 |  |
| Kitchen location | Low: No kitchen, in bedroom | 42.2 | 26.6 | 10 | 8.3 | 8.7 | 4.7 | 2.9 |  |
|  | Medium: In separate place | 57.8 | 52.1 | 62.2 | 24.3 | 38.2 | 31.1 | 24.7 |  |
|  | High: Built-in housing |  | 21.3 | 27.7 | 67.4 | 53.2 | 64.2 | 72.4 |  |
| Roof quality | Low: Thatched or similar material | 27.1 | 27.9 | 11 | 5.7 | 2.4 | 0.8 | 0.9 |  |
|  | Medium: Tile, metal | 72.9 | 72.1 | 89 | 94.3 | 94.1 | 78.5 | 78.8 |  |
|  | High: Concrete, duralite |  |  |  |  | 3.4 | 20.7 | 20.3 |  |
| Sewage facility | Low: No drain | 98.9 | 99.2 | 99.7 | 99.1 | 89.3 | 46 | 45.3 |  |
|  | Medium: Cesspit system | 1.1 | 0.8 | 0.2 | 0.8 |  | 2 | 0.2 |  |
|  | High: Sewage system, septic tank |  |  | 0.2 | 0.2 | 10.7 | 52 | 54.5 |  |
| Stove quality | Low: None, on floor | 98.5 | 94.7 | 96.3 | 53.2 | 45.6 | 32.2 | 26.6 |  |
|  | Medium: Low or high removable/fixed wood and charcoal stove | 1.5 |  | 1.5 | 43.7 | 54.4 | 66.7 | 72.3 |  |
|  | High: Gas or electric stove |  | 5.3 | 2.3 | 3.1 |  | 1.1 | 1.1 |  |
| Toilet quality | Low: None | 94.5 | 94.2 | 100 | 97.5 | 81 | 20 | 23.4 |  |
|  | Medium: Latrine, pit latrine | 5.5 | 5.8 |  | 2.5 | 12.4 | 22.9 | 19.3 |  |
|  | High: Septic tank + indoor toilet |  |  |  |  | 6.6 | 57.1 | 57.3 |  |
| Wall quality | Low: Cane or similar, mix of clay, cane and wood (with or without cement) | 54.3 | 43.7 | 18.6 | 12.6 | 7.4 | 4.6 | 2.3 |  |
|  | Medium: Mud brick with or without cement, wood, metal | 45.7 | 56.3 | 72.6 | 68.2 | 52.9 | 24.2 | 20.3 |  |
|  | High: Brick |  |  | 8.8 | 19.2 | 39.6 | 71.3 | 77.5 |  |
| Source of water quality | Low: Spring or river | 51.9 | 18.1 | 3.1 | 1.2 |  | 2.1 | 2.2 |  |
|  | Medium: Public pool with pitchers, well in neighborhood or house | 48.1 | 81.9 | 63.7 | 8.6 | 4.3 | 6 | 9.1 |  |
|  | High: Public water system |  |  | 33.2 | 90.2 | 95.7 | 91.8 | 88.7 |  |
| Land ownership | Yes, No |  | 73.5 | 80.4 | 72.8 | 53.1 | 72.9 | 67.1 |  |
| Birds | Yes, No |  |  | 64.5 | 62 | 57.2 | 41.9 | 28.6 |  |
| Pigs | Yes, No |  |  | 48.5 | 29.7 | 25.2 | 9 | 7.9 |  |
| Turntable | Yes, No |  | 2.4 | 6 | 12.6 |  |  |  |  |
| Hand grinder | Yes, No |  |  | 8.1 | 7.2 | 4.8 |  |  |  |
| Electric iron | Yes, No |  |  | 32.9 | 79.5 | 83.9 |  |  |  |
| Cassette player | Yes, No |  |  |  | 55.2 | 38.2 |  |  |  |
| Sound system | Yes, No |  |  |  |  | 37.9 | 59 | 57.5 |  |
| Video player | Yes, No |  |  |  | 4.5 | 8.7 | 55.2 | 47.5 |  |
| Cable | Yes, No |  |  |  |  | 5 | 63.6 | 67.4 |  |
| Blender | Yes, No |  |  |  |  | 41.2 | 77.2 | 82.6 |  |
| Typewriter | Yes, No |  |  |  |  | 9.9 |  |  |  |
| Garbage disposal quality | Low: Throw in yard |  |  |  |  |  | 1.5 | 0.8 |  |
|  | Medium: Bury, burn or throw in ravine |  |  |  |  |  | 50 | 50.4 |  |
|  | High: Public dump |  |  |  |  |  | 48.5 | 48.7 |  |
| Microwave | Yes, No |  |  |  |  | 2.4 | 32.8 | 32.8 |  |
| Computer | Yes, No |  |  |  |  | 1.3 | 34.3 | 30.4 |  |
| Cellphone | Yes, No |  |  |  |  |  | 94.9 | 95.3 |  |
| Ipod | Yes, No |  |  |  |  |  | 10.1 | 12.8 |  |
| Washing machine | Yes, No |  |  |  |  |  | 18.7 | 20.7 |  |
| Internet | Yes, No |  |  |  |  |  |  | 13.2 |  |
| Direct TV | Yes, No |  |  |  |  |  |  | 5.3 |  |

# Supplementary Table 1C. Categorization and availability of assets for New Delhi Birth Cohort by study wave

|  | **Survey Year** |  |  |  |  |  |  |  |  |
| --- | --- | --- | --- | --- | --- | --- | --- | --- | --- |
|  | ***Percentage of original sample with asset data*** | 74.6% | 18.7% | 14.0% | 9.7% | 10.3% |  |  |  |
| **Asset** | **Categorization** | **1969-72** | **1998-02** | **2006-09** | **2012-16** | **2016-19** |  |  |  |
| Air conditioner | Yes, No |  | 29.6 | 50.8 | 72.2 | 80.9 |  |  |  |
| Bed | Yes, No |  |  |  |  | 99.9 |  |  |  |
| Bicycle | Yes, No |  | 28.1 | 58.6 | 56 | 50.3 |  |  |  |
| Cable TV | Yes, No |  | 93.1 | 87.5 | 55.6 |  |  |  |  |
| Car | Yes, No |  | 45.1 | 59.7 | 68.8 | 69.9 |  |  |  |
| Cellphone | Yes, No |  |  | 95.7 | 99.6 | 99.3 |  |  |  |
| Chair | Yes, No |  |  |  |  | 99.8 |  |  |  |
| Clock | Yes, No |  |  |  |  | 100 |  |  |  |
| Computer | Yes, No |  | 17.6 | 45.6 | 78 | 79.5 |  |  |  |
| Cooler | Yes, No |  | 91.2 | 85.3 | 61.7 | 48.5 |  |  |  |
| Dish TV | Yes, No |  | 1.4 | 14.6 | 56.5 |  |  |  |  |
| Electricity | Yes, No |  | 99.9 | 100 | 100 | 99.9 |  |  |  |
| Fan | Yes, No |  | 100 | 99.8 | 99.9 | 100 |  |  |  |
| Internet | Yes, No |  |  |  |  | 77.1 |  |  |  |
| Mattress | Yes, No |  |  |  |  | 100 |  |  |  |
| Mixer grinder | Yes, No |  | 90.8 | 94.4 | 95.2 |  |  |  |  |
| Owns house | Yes, No |  |  |  |  | 97.6 |  |  |  |
| Pressure cooker | Yes, No |  |  |  |  | 99.9 |  |  |  |
| Radio | Yes, No |  | 88.7 | 80.7 | 49.4 | 44.4 |  |  |  |
| Refrigerator | Yes, No |  |  |  |  | 99 |  |  |  |
| Separate kitchen | Yes, No |  |  |  |  | 98.9 |  |  |  |
| Sewing machine | Yes, No |  |  |  |  | 61.1 |  |  |  |
| Stove | Yes, No |  | 98.6 | 99.1 | 99.5 |  |  |  |  |
| Table | Yes, No |  |  |  |  | 99.6 |  |  |  |
| Telephone | Yes, No |  | 86.2 | 73.2 | 48.7 | 48.9 |  |  |  |
| Television (any) | Yes, No |  | 99.6 | 99.3 | 99.2 | 98.9 |  |  |  |
| Television – black & white | Yes, No |  |  |  |  | 2.3 |  |  |  |
| Television – color | Yes, No |  |  |  |  | 77.7 |  |  |  |
| Television – plasma | Yes, No |  |  |  |  | 76.1 |  |  |  |
| Tractor | Yes, No |  |  |  |  | 0.5 |  |  |  |
| Two wheeler | Yes, No |  | 81.4 | 77.7 | 77 | 74.8 |  |  |  |
| Washing machine | Yes, No |  | 79.2 | 89.4 | 94.1 |  |  |  |  |
| Water pump | Yes, No |  |  |  |  | 77.8 |  |  |  |
| Drinking water | Low: Unprotected, Open well/river |  |  | 1.6 | 0.1 | 0.2 |  |  |  |
|  | Medium: Both protected and unprotected, borewell or handpump |  | 6 | 3.4 | 7.8 |  |  |  |  |
|  | High: Protected, tap, mineral water, tanker water |  | 94 | 95 | 92 | 99.8 |  |  |  |
| Sharing of drinking water | Low: Communal | 17.7 | 0.7 | 0.1 | 1.6 |  |  |  |  |
|  | Medium: Common | 47.8 | 10.3 | 5.7 | 20.4 |  |  |  |  |
|  | High: Separate, mineral water, tanker water | 34.4 | 88.9 | 94.2 | 78 |  |  |  |  |
| General water supply | Low: Unprotected, Open well/river |  |  | 0.9 |  |  |  |  |  |
|  | Medium: Both protected and unprotected, borewell or handpump |  | 14.5 | 5.2 | 17.2 |  |  |  |  |
|  | High: Protected, tap, mineral water, tanker water |  | 85.5 | 93.9 | 82.8 |  |  |  |  |
| Sharing of general water | Low: Communal |  | 0.4 | 0.2 | 1.4 |  |  |  |  |
|  | Medium: Common |  | 6.7 | 10.5 | 28.5 |  |  |  |  |
|  | High: Separate, mineral water, tanker water |  | 92.9 | 89.3 | 70.1 |  |  |  |  |
| Type of house | Low: Thatched hut | 1.8 |  |  |  |  |  |  |  |
|  | Medium: Masonry built | 67.2 | 0.2 | 0.4 | 0.1 |  |  |  |  |
|  | High: Flats, Bungalow, Other | 31 | 99.8 | 99.6 | 99.9 |  |  |  |  |
| Type of lighting | Low: Oil |  |  |  |  |  |  |  |  |
|  | Medium: Kerosene, gas |  |  |  |  | 0.1 |  |  |  |
|  | High: Electricity |  |  |  |  | 99.9 |  |  |  |
| Type of toilet | Low: Open field |  | 0.7 |  | 0.1 | 0.2 |  |  |  |
|  | Medium:  1969-72: Pit, scavenger cleaned |  | 10.3 | 3.7 | 3.3 | 0.4 |  |  |  |
|  | High: Flush |  | 89 | 96.3 | 96.6 | 99.4 |  |  |  |
| Sharing of toilet | Low: Communal | 22.7 |  |  |  | 99.3 |  |  |  |
|  | Medium: Common | 39.8 |  |  |  | 0.7 |  |  |  |
|  | High: Separate | 37.5 |  |  |  |  |  |  |  |

# Supplementary Table 1D. Categorization and availability of assets for Cebu Longitudinal Health and Nutrition Study by study wave

|  | **Survey Year** |  |  |  |  |  |  |  |  |
| --- | --- | --- | --- | --- | --- | --- | --- | --- | --- |
|  | ***Percentage of original sample with asset data*** | 100% | 72.7% | 71.1% | 67.8% | 65.6% | 61.3% | 55.6% | 43.1% |
| **Asset** | **Categorization** | **1983** | **1991** | **1994** | **1998** | **2002** | **2005** | **2009** | **2018** |
| Air conditioning | Yes, No | 0.3 | 0.7 | 1.0 | 2.8 | 4.4 | 6.7 | 9.9 | 19.7 |
| Bicycle | Yes, No | 14.5 | 17.8 | 23 | 25.7 | 29.3 | 29.4 | 23.8 | 29.1 |
| Car | Yes, No | 0.8 | 1.5 | 2.3 | 3.4 | 3.1 | 4.2 | 4.4 | 9.5 |
| Chicken/poultry | Yes, No | 42.8 | 49.3 | 50 | 46.5 | 50.5 | 42.2 | - | 39.2 |
| Electric fan | Yes, No | 14.3 | 37.5 | 45.8 | 59.4 |  | 73.2 | 78.3 | 88.3 |
| Electric iron | Yes, No | 9.8 | 28.9 | 37.9 | 50.9 | 58 | 58.1 | 56.6 | 60.9 |
| Electricity | Yes, No |  | 73.8 | 80.1 | 87.2 | 91.8 | 94.4 | 95 | 96.1 |
| Jepny | Yes, No | 0.9 | 1.7 | 2.2 | 3 | 5.2 | 6.6 | 6.5 | 6.8 |
| Living room set | Yes, No | 27 | 41 | 42.8 | 47.1 | 52.4 | 52.4 | - | 46.6 |
| Other appliances | Yes, No | 2.8 | 8.9 | 50.3 | 65.9 | 22.3 | 6 | 4.7 | - |
| House | Yes, No | 65.9 | 84 | 86.5 | 88.5 | 87 | 84.4 | 78.5 | 80 |
| Refrigerator | Yes, No | 6.7 | 21.7 | 28.5 | 37 | 41.3 | 42.3 | 42.1 | 47.2 |
| Cleanliness of are where food is stored | Low: Filthy | 8.8 | 15.6 | 6.7 | 20.3 | 14.3 | 7.2 | 8 | 14.2 |
|  | Medium: Not so clean | 72.8 | 61.7 | 75.7 | 68.3 | 63.5 | 69.5 | 68.5 | 49.3 |
|  | High: Very clean | 18.5 | 22.7 | 17.6 | 11.5 | 22.2 | 23.3 | 23.5 | 36.5 |
| Cooking fuel | Low: Wood/charcoal, sawdust, other | 83.9 | 61.8 | 47.5 | 33.6 | 36.1 | 44.7 | 60.5 | 17.1 |
|  | Medium: Kerosene, combination of fuels | 10 | 22.1 | 29.8 | 29.4 | 18.9 | 17.3 | 5 | 0.2 |
|  | High: Electricity, LPG | 6.1 | 16.1 | 22.7 | 36.9 | 45 | 38 | 34.5 | 82.7 |
| Garbage disposal | Low: Dumped around/near house, in stream or river, combination of methods, Dumped away from house | 42.5 | 47.3 | 48.5 | 55.7 | 10.5 | 9.5 | 14.2 | 6.3 |
|  | Medium: Burning, other | 41 | 52.7 | 51.5 | 44.3 | 35.6 | 35.7 | 21.4 | 16.2 |
|  | High: Collected by garbage collector, composting | 16.5 |  |  |  | 53.9 | 54.8 | 64.4 | 77.5 |
| Condition of area for excreta | Low: Heavy defecation in area | 6.5 | 11.8 | 6.4 | 20.6 | 11.6 | 5.5 | 3.6 | 1.7 |
|  | Medium: Some defecation or very little defecation in area | 43.9 | 57.6 | 78.2 | 73 | 63.4 | 69.1 | 57.6 | 35.2 |
|  | High: No excreta visible | 49.5 | 30.6 | 15.4 | 6.5 | 25.1 | 25.4 | 38.8 | 63.1 |
| Lighting | Low: Oil, Candle | 0.1 |  | 0 | 0 | 0.1 | 0.3 | 0.1 | 0.4 |
|  | Medium: Kerosene, Other material | 50.1 | 26.5 | 19.9 | 13.9 | 9.1 | 7 | 6.4 | 4.9 |
|  | High: Electricity, LPG | 49.7 | 73.5 | 80 | 86 | 90.7 | 92.8 | 93.6 | 94.7 |
| Housing material | Low: Wood or similar | 43 | 40.2 | 38 | 25.9 | 23.1 | 23.5 | 19.7 | 30.9 |
|  | Medium: Cement and/or wood mixed with similar roofing | 38.9 | 39.2 | 50.1 | 60.7 | 49.6 | 56.1 | 54.7 | 39.3 |
|  | High: Cement or wood with galvanized iron roofing | 18.1 | 20.6 | 11.9 | 13.4 | 27.3 | 20.4 | 25.6 | 29.8 |
| Neighborhood excreta removal | Low: Heavy defecation in area | - | 10.9 | 7 | 18.6 | 12 | 6.3 | 4.3 | 4.4 |
|  | Medium: Some defecation or very little defecation in area | - | 64.8 | 85 | 77.6 | 71.8 | 73.4 | 67.5 | 44.6 |
|  | High: No excreta visible | - | 24.2 | 7.9 | 3.8 | 16.2 | 20.3 | 28.2 | 50.9 |
| Neighborhood garbage removal | Low: Lots of uncollected garbate | - | 12.6 | 5.1 | 15.2 | 11.6 | 6.4 | 5.1 | 14.3 |
|  | Medium: Some or very little garbage | - | 80.7 | 91.1 | 83.7 | 82.1 | 85.6 | 77.4 | 46.8 |
|  | High: No garbage visible | - | 6.6 | 3.8 | 1.1 | 6.3 | 8 | 17.5 | 38.9 |
| Drinking water | Low: Spring, river, rainwater | 6.6 | 8.9 | 8.7 | 10 | 8.2 | 6.9 | 27.3 | 4.4 |
|  | Medium: Dug well without pump, open well | 80.1 | 12.7 | 46 | 29.7 | 16.3 | 20.4 | 10.6 | 0.2 |
|  | High: Metro or other piped supply, Tubewell/motorized pump, purchased mineral/bottled water | 13.4 | 78.3 | 45.3 | 60.3 | 75.5 | 72.8 | 62.1 | 95.4 |
| Toilet | Low: None or other | 28.8 | 33.5 | 28 | 22.9 | 18.5 | 14.7 | 11.3 | 5.2 |
|  | Medium: Latrine, open pit | 31.4 | 9.3 | 7.2 | 4.2 | 2.9 | 3 | 1.3 | 0.9 |
|  | High: Flush toilet (inside or outside), water-sealed toilet (inside or outside) | 39.8 | 57.2 | 64.7 | 72.9 | 78.6 | 82.3 | 87.3 | 93.9 |
| Beds | Yes, No | 38.8 | 43 | 51.8 | 65.5 | 68.6 | 71.7 | - | 71.3 |
| Boat | Yes, No | 0.3 |  | 2 | 2.3 | 1.2 | 0.8 | 1 | 0.8 |
| Cattle (cows or carabaos) | Yes, No | 5.7 | 7.9 | 8 | 6.7 | 6 | 4.5 | - | 3 |
| Farm animals (goat, horse, pig etc) | Yes, No | 36.7 | 33.1 | 24.7 | 18.1 | 20.2 | 15.8 | - | 7.9 |
| Other vehicles (banca, motorcycle or tricycle with side-car etc) | Yes, No | 5.3 | 3.4 | 0.6 | 2.3 | 8.3 | 8.2 | 7.9 | 8 |
| Truck or bus | Yes, No | 0.1 | 0.3 | 0.4 | 0.9 | 0.7 | 0.7 | 0.8 | 0.8 |
| Television | Yes, No | 18.2 | 53.7 | 61.9 | 70.8 | 62.4 | 72.9 | 78 | 76.6 |
| Drinking water storage | Low: Open drum, can (tin) | 1.6 |  |  |  |  |  |  |  |
|  | Medium: Earthern jar or plastic container without faucet | 65 |  |  |  |  |  |  |  |
|  | High: Container in fridge, water tank, earthern jar or plastic container with faucet | 33.4 |  |  |  |  |  |  |  |
| Beautician kit | Yes, No | 8.9 |  |  |  |  |  |  |  |
| Benches or chairs | Yes, No | 68.6 | 74.8 |  |  |  |  |  |  |
| Bottle brush | Yes, No | 23.7 |  |  |  |  |  |  |  |
| Chest/closet of drawers | Yes, No | 68.1 | 58.3 | 61.3 | 67.2 |  |  |  |  |
| Clay pots/ pan | Yes, No | 75.6 |  |  |  |  |  |  |  |
| Clay stove | Yes, No | 53.9 |  |  |  |  |  |  |  |
| Dining set | Yes, No | 13.2 | 15.1 |  |  | 59.1 | 67.6 |  | 51.8 |
| Feeding bottles | Yes, No | 44.8 |  |  |  |  |  |  |  |
| Flat iron | Yes, No | 42.9 |  |  |  |  |  |  |  |
| Glassware | Yes, No | 41 |  |  |  |  |  |  |  |
| Kerosene stove | Yes, No | 16.9 |  |  |  | 32.2 | 27.2 | 10.9 | 3.2 |
| Measuring spoon | Yes, No | 8.4 |  |  |  |  |  |  |  |
| Other business machine | Yes, No | 6.5 |  |  |  |  |  |  |  |
| Other kitchen equipment | Yes, No | 67.7 |  |  |  |  |  |  |  |
| Other agricultural equipment | Yes, No | 20.7 |  |  |  |  |  |  |  |
| Radio | Yes, No | 55.8 |  |  |  |  |  |  |  |
| Sewing machine | Yes, No | 12.5 |  |  |  | 20.1 | 17.5 | 14.8 | 9.7 |
| Tables | Yes, No | 69.9 | 76.7 |  |  |  |  |  |  |
| Tape recorder/ stereo set | Yes, No | 18.8 | 44.1 |  |  |  | 35.6 | 34.4 | 15.6 |
| Thermos bottle | Yes, No | 56.3 |  |  |  |  |  |  |  |
| Other house | Yes, No | 7.9 |  |  |  |  |  |  |  |
| Gas stove | Yes, No | 12.3 |  |  |  | 38.9 | 38.9 | 44.5 | 80 |
| Other furniture | Yes, No | 0.6 | 8.7 | 19.3 | 84.5 | 27 | 5.2 |  |  |
| Electricity in neighborhood | Yes, No |  | 87.7 | 91.9 | 94.3 | 97.8 | 98.9 |  | 99.4 |
| Neighborhood construction material | Low: Light (bamboo, nipa, cheap wood) |  |  | 39.9 | 20 | 21.4 | 20.3 | 16.3 | 18.6 |
|  | Medium: Mixed (wood with hollow blocks, cement) |  |  | 54.5 | 74.8 | 61.9 | 63.1 | 63.2 | 62.6 |
|  | High: Strong (hollow blocks, concrete or good wood) |  |  | 5.6 | 5.2 | 16.7 | 16.6 | 20.5 | 18.8 |
| China cabinet | Yes, No |  |  | 7.9 | 14.9 | 75.9 | 75.5 |  | 73.7 |
| Motorcycle | Yes, No |  |  | 5.2 | 8.4 | 12.2 | 16.3 | 26.1 | 49.2 |
| Digital camera | Yes, No |  |  | 16.2 | 27.7 | 40.1 | 11.6 | 19.1 | 18.5 |
| Phone | Yes, No |  |  | 6.4 | 18 | 40.4 | 68 | 40.7 | 93.3 |
| House is neat | Low: Poorly kept, dirty or messy |  |  |  | 21.6 | 16.7 | 10.5 | 9.7 | 20.1 |
|  | Medium: Not so neat |  |  |  | 66.7 | 63 | 68 | 69.2 | 44.2 |
|  | High: Neat and tidy |  |  |  | 11.7 | 20.3 | 21.5 | 21.1 | 35.7 |
| Cable + TV | Yes, No |  |  |  |  | 6.9 | 6.7 | 5.4 | 12.4 |
| CD player | Yes, No |  |  |  |  | 18.8 | 31.2 | 46.7 | 59.2 |
| Karaoke machine | Yes, No |  |  |  |  | 45.3 | 45.9 | 42.6 | 46.6 |
| Oven | Yes, No |  |  |  |  | 17.6 | 14.7 | 12.3 | 11 |
| Pressure cooker | Yes, No |  |  |  |  | 15.7 | 16.3 | 15.8 | 26.9 |
| Rice cooker | Yes, No |  |  |  |  | 22 | 29.9 | 44.7 | 54.4 |
| VCR player | Yes, No |  |  |  |  | 41.9 | 51 | 68.1 |  |
| Washing machine | Yes, No |  |  |  |  | 20.5 | 22.8 | 23.7 | 37.5 |
| Computer | Yes, No |  |  |  |  | 5.1 | 11.1 | 19.7 | 27.7 |
| Microwave | Yes, No |  |  |  |  | 3.5 | 6 | 11.7 |  |
| Video game | Yes, No |  |  |  |  |  | 8.2 | 9.1 | 6.9 |
| Non-drinking water source | Low: Spring, River, Rainwater |  |  |  |  |  |  |  | 8.2 |
|  | Medium: Dug well without pump |  |  |  |  |  |  |  | 15 |
|  | High: Piped supply (Metro, other), Tubewell or borehole or motorized pump |  |  |  |  |  |  |  | 76.8 |
| Vacuum cleaner | Yes, No | 0.1 |  |  |  | 2.4 | 2.7 | 3.8 | 6.4 |
| Tablet | Yes, No |  |  |  |  |  |  |  | 32.7 |
| Electric water dispenser | Yes, No |  |  |  |  |  |  |  | 14.1 |

# Supplementary Table 1E. Categorization and availability of assets for Birth to Twenty plus cohort by study wave

|  | **Survey Year** |  |  |  |  |  |  |  |  |
| --- | --- | --- | --- | --- | --- | --- | --- | --- | --- |
|  | ***Percentage of original sample with asset data*** | 85.9% | 41.1% | 44.1% | 46.2% | 50.0% | 42.6% |  |  |
| **Asset** | **Categorization** | **1990-92** | **1997-98** | **2002-03** | **2006-07** | **2012-13** | **2017-18** |  |  |
| Car | Yes, No | 32.4 | 27 | 28.5 | 32.4 | 42.3 | 44 |  |  |
| Electricity | Yes, No | 92.1 | 97.2 | 96.9 | 97.5 | 95.8 |  |  |  |
| Microwave | Yes, No |  | 16.7 | 38.1 | 58.8 | 82 | 83.2 |  |  |
| Radio | Yes, No | 84.5 | 87.9 | 86.9 | 87.9 | 88.1 |  |  |  |
| Refrigerator | Yes, No | 70.4 | 87.4 | 91 | 93.6 | 93.2 | 92.7 |  |  |
| Telephone | Yes, No | 53.5 | 51.5 | 50.5 | 40.4 | 25.8 | 42.4 |  |  |
| Television | Yes, No | 73.7 | 92.1 | 92.1 | 95.4 | 94.6 | 92.4 |  |  |
| Washing machine | Yes, No | 20.1 | 26.9 | 33.4 | 46.5 | 66.9 | 72.1 |  |  |
| Indoor flush toilet | Yes, No | 36.9 | 38.5 | 49.5 | 54.1 |  | 73.5 |  |  |
| Indoor hot or cold water | Yes, No | 54.9 | 55.7 | 67.7 | 75.0 |  | 78.6 |  |  |
| Housing type | Low: Shack |  | 6.3 | 8.4 |  |  | 6.6 |  |  |
|  | Medium: Room, Shared house, Hostel, Garage | 17.9 | 5.5 | 6.1 |  |  | 11.6 |  |  |
|  | High: House, Flat | 82.1 | 88.2 | 85.5 |  |  | 81.8 |  |  |
| Refuse disposal | Low: Leave in the street, other |  |  |  |  |  |  |  |  |
|  | Medium: Communal heap, own refuse heap |  |  |  |  |  |  |  |  |
|  | High: Own garbage bin |  |  |  |  |  |  |  |  |
| House ownership | Yes, No | 25.2 | 27.2 |  |  |  | 78.5 |  |  |
| Solo usage of water | Yes, No | 76.6 |  | 76.1 | 70.9 |  |  |  |  |
| Solo usage of toilet | Yes, No | 74.8 |  | 87 | 46.4 |  |  |  |  |
| DVD player | Yes, No |  | 30 | 39.9 | 68.1 | 86.1 |  |  |  |
| Cellphone | Yes, No |  |  | 58.9 | 91.4 | 95.1 | 88.4 |  |  |
| Mnet | Yes, No |  |  | 4.2 | 6.7 |  |  |  |  |
| Satellite TV | Yes, No |  |  | 3.1 | 7.2 |  |  |  |  |
| Computer | Yes, No |  |  |  | 21.8 | 58 | 57.6 |  |  |
| Internet | Yes, No |  |  |  | 4 | 57.5 | 53.4 |  |  |

# Supplementary Table 2A. Comparison of early life characteristics for Pelotas 1993 cohort for non-participants in study wave

|  | **Original** | **Not available or Died** | | | | |  |  |  |
| --- | --- | --- | --- | --- | --- | --- | --- | --- | --- |
|  | **1993** | **1997** | **2004** | **2008** | **2011** | **2015** |  |  |  |
| **N** | **5249** | **3976** | **822** | **909** | **1142** | **1438** |  |  |  |
| Maternal age | 26.0±6.4 | 25.9±6.4 | 25.8±6.4 | 25.6±6.4 | 25.7±6.5 | 25.9±6.5 |  |  |  |
| Paternal age | 29.5±7.7 | 29.5±7.8 | 29.2±7.4 | 29.3±7.5 | 29.2±7.6 | 29.4±7.8 |  |  |  |
| Maternal education | 6.7±3.6 | 6.8±3.6 | 7.1±4.0 | 6.8±3.9 | 6.6±3.9 | 6.4±3.7 |  |  |  |
| Paternal education | 6.8±3.5 | 6.9±3.6 | 7.2±3.9 | 7.0±3.9 | 6.9±3.8 | 6.6±3.6 |  |  |  |
| Mother employed during pregnancy |  |  |  |  |  |  |  |  |  |
| Yes | 1911 (36.4%) | 1447 (36.4%) | 302 (36.8%) | 316 (34.8%) | 382 (33.5%) | 478 (33.3%) |  |  |  |
| No | 3229 (61.6%) | 2453 (61.7%) | 496 (60.5%) | 567 (62.5%) | 737 (64.6%) | 934 (65.0%) |  |  |  |
| Student | 73 (1.4%) | 55 (1.4%) | 17 (2.1%) | 20 (2.2%) | 18 (1.6%) | 16 (1.1%) |  |  |  |
| Stay at home | 31 (0.6%) | 18 (0.5%) | 5 (0.6%) | 4 (0.4%) | 4 (0.4%) | 8 (0.6%) |  |  |  |
| Maternal skin color |  |  |  |  |  |  |  |  |  |
| White | 4058 (77.4%) | 3081 (77.5%) | 653 (79.4%) | 725 (79.8%) | 903 (79.1%) | 1148 (79.8%) |  |  |  |
| Black | 954 (18.2%) | 724 (18.2%) | 140 (17.0%) | 151 (16.6%) | 191 (16.7%) | 234 (16.3%) |  |  |  |
| Other | 234 (4.5%) | 169 (4.3%) | 29 (3.5%) | 33 (3.6%) | 48 (4.2%) | 56 (3.9%) |  |  |  |
| Sex |  |  |  |  |  |  |  |  |  |
| Female | 2645 (50.4%) | 1981 (49.8%) | 399 (48.5%) | 431 (47.4%) | 554 (48.5%) | 618 (43.0%) |  |  |  |
| Male | 2603 (49.6%) | 1995 (50.2%) | 423 (51.5%) | 478 (52.6%) | 588 (51.5%) | 820 (57.0%) |  |  |  |
| Skin color of index child |  |  |  |  |  |  |  |  |  |
| White | 2769 (64.1%) | 2062 (64.6%) | 68 (70.1%) | 3 (60.0%) | 243 (62.5%) | 507 (67.7%) |  |  |  |
| Black | 611 (14.1%) | 437 (13.7%) | 6 (6.2%) | 0 (0.0%) | 43 (11.1%) | 73 (9.7%) |  |  |  |
| Other | 943 (21.8%) | 692 (21.7%) | 23 (23.7%) | 2 (40.0%) | 103 (26.5%) | 169 (22.6%) |  |  |  |

# Supplementary Table 2B. Comparison of early life characteristics for INCAP Longitudinal Study cohort for participants in study wave

|  |  | **Not available or Died** | | | | |  |
| --- | --- | --- | --- | --- | --- | --- | --- |
|  | **Original** | **1987** | **1996** | **2002** | **2015-16** | **2017-18** |  |
| ***N*** | 2392 | 1032 | 1539 | 1339 | 1229 | 1127 |  |
| Maternal age at birth of index child | 27.0±7.2 | 26.4±7.2 | 26.4±7.2 | 26.5±7.2 | 27.0±7.3 | 27.0±7.4 |  |
| Maternal height (cm) | 148.7±5.2 | 148.4±5.4 | 148.7±5.2 | 148.7±5.2 | 148.9±5.4 | 149.0±5.4 |  |
| Maternal schooling | 1.0 [0.0;2.0] | 0.0 [0.0;2.0] | 1.0 [0.0;2.0] | 1.0 [0.0;2.0] | 1.0 [0.0;2.0] | 1.0 [0.0;2.0] |  |
| Birth year (19XX) | 71 [67;74] | 69 [66;72] | 71 [67;73] | 71 [67;74] | 71 [67;74] | 71 [67;74] |  |
| Type of nutritional supplementation |  |  |  |  |  |  |  |
| Fresco supplementation | 1123 (46.9%) | 470 (45.5%) | 681 (44.2%) | 602 (45.0%) | 592 (48.2%) | 525 (46.6%) |  |
| Atole supplementation | 1269 (53.1%) | 562 (54.5%) | 858 (55.8%) | 737 (55.0%) | 637 (51.8%) | 602 (53.4%) |  |
| Sex |  |  |  |  |  |  |  |
| Male | 1230 (51.4%) | 471 (45.6%) | 787 (51.1%) | 670 (50.0%) | 766 (62.3%) | 668 (59.3%) |  |
| Female | 1162 (48.6%) | 561 (54.4%) | 752 (48.9%) | 669 (50.0%) | 463 (37.7%) | 459 (40.7%) |  |

# Supplementary Table 2C. Comparison of early life characteristics for New Delhi Birth Cohort for participants in study wave

|  |  | **Not available or Died** | | | | |  |  |
| --- | --- | --- | --- | --- | --- | --- | --- | --- |
|  | **Original** | **1969-72** | **1998-02** | **2006-09** | **2012-16** | **2016-19** |  |  |
| N | 8181 | 2068 | 6643 | 7030 | 7383 | 7335 |  |  |
| Maternal age at birth of index child | 25.9±5.2 | 29.3±0.6 | 25.7±5.1 | 25.8±5.1 | 25.8±5.2 | 25.8±5.2 |  |  |
| Maternal schooling | 3.0 [0.0;10.0] | 10.0 [3.0;12.0] | 3.0 [0.0;10.0] | 3.0 [0.0;10.0] | 3.0 [0.0;10.0] | 3.0 [0.0;10.0] |  |  |
| paternal education | 12.0 [8.0;15.0] | 12.0 [12.0;15.0] | 12.0 [8.0;13.5] | 12.0 [8.0;15.0] | 12.0 [8.0;15.0] | 12.0 [8.0;15.0] |  |  |
| year of birth | 1971.0 [1970.0;1972.0] | 1971.0 [1970.0;1972.0] | 1971.0 [1970.0;1972.0] | 1971.0 [1970.0;1972.0] | 1971.0 [1970.0;1972.0] | 1971.0 [1970.0;1972.0] |  |  |
| Sex |  |  |  |  |  |  |  |  |
| Male | 3924 (48.0%) | 1074 (51.9%) | 3036 (45.7%) | 3265 (46.4%) | 3436 (46.5%) | 3405 (46.4%) |  |  |
| Female | 3606 (44.1%) | 991 (47.9%) | 2966 (44.6%) | 3134 (44.6%) | 3315 (44.9%) | 3299 (45.0%) |  |  |
| 'Missing' | 641 (7.8%) | 3 (0.1%) | 641 (9.6%) | 631 (9.0%) | 632 (8.6%) | 631 (8.6%) |  |  |
| Religion |  |  |  |  |  |  |  |  |
| Hindu | 5172 (63.3%) | 6 (0.3%) | 4263 (64.2%) | 4491 (63.9%) | 4709 (63.8%) | 4676 (63.7%) |  |  |
| Muslim | 81 (1.0%) | 0 (0.0%) | 80 (1.2%) | 80 (1.1%) | 81 (1.1%) | 81 (1.1%) |  |  |
| Sikh | 651 (8.0%) | 0 (0.0%) | 523 (7.9%) | 550 (7.8%) | 586 (7.9%) | 589 (8.0%) |  |  |
| Jain | 47 (0.6%) | 0 (0.0%) | 32 (0.5%) | 37 (0.5%) | 36 (0.5%) | 36 (0.5%) |  |  |
| Christian | 142 (1.7%) | 0 (0.0%) | 138 (2.1%) | 140 (2.0%) | 140 (1.9%) | 141 (1.9%) |  |  |
| Others | 12 (0.1%) | 0 (0.0%) | 12 (0.2%) | 12 (0.2%) | 12 (0.2%) | 11 (0.1%) |  |  |
| 'Missing' | 2066 (25.3%) | 2062 (99.7%) | 1595 (24.0%) | 1720 (24.5%) | 1819 (24.6%) | 1801 (24.6%) |  |  |

# Supplementary Table 2D. Comparison of early life characteristics for Cebu Longitudinal Health and Nutrition Study for participants in study waves

|  |  | **Not available or Died** | | | | | | |
| --- | --- | --- | --- | --- | --- | --- | --- | --- |
| **Categorization** | **Original** | **1991** | **1994** | **1998** | **2002** | **2005** | **2009** | **2018** |
| ***N*** | 3080 | 816 | 894 | 998 | 1065 | 1194 | 1371 | 1754 |
| Maternal age at birth of index child | 26.3±6.0 | 26.2±5.8 | 26.2±5.9 | 26.2±6.0 | 26.2±6.0 | 26.2±6.0 | 26.1±6.0 | 26.1±5.9 |
| Maternal schooling (y) | 6.0 [5.0;9.0] | 6.0 [5.0;10.0] | 6.0 [5.0;10.0] | 6.0 [5.0;10.0] | 6.0 [5.0;10.0] | 6.0 [5.0;10.0] | 6.0 [5.0;10.0] | 6.0 [5.0;10.0] |
| Maternal height (cm) | 150.6±5.0 | 150.5±5.0 | 150.5±5.0 | 150.5±5.0 | 150.5±5.1 | 150.6±5.0 | 150.7±5.0 | 150.8±5.1 |
| Sex |  |  |  |  |  |  |  |  |
| Male | 1632 (53.0%) | 437 (53.6%) | 487 (54.5%) | 545 (54.6%) | 564 (53.0%) | 640 (53.6%) | 739 (53.9%) | 917 (52.3%) |
| Female | 1448 (47.0%) | 379 (46.4%) | 407 (45.5%) | 453 (45.4%) | 501 (47.0%) | 554 (46.4%) | 632 (46.1%) | 837 (47.7%) |

# Supplementary Table 2E. Comparison of early life characteristics for Birth to Twenty plus cohort for participants in study waves

|  |  | **Not available or Died** | | | | |  |  |
| --- | --- | --- | --- | --- | --- | --- | --- | --- |
| **Categorization** | **Original** | **1997-98** | **2002-03** | **2006-07** | **2012-13** | **2017-18** |  |  |
| ***N*** | 3273 | 1928 | 1831 | 1762 | 1650 | 1879 |  |  |
| Maternal age (y) | 26.0±6.1 | 26.2±6.0 | 26.0±5.9 | 26.1±5.9 | 26.1±5.9 | 26.1±5.9 |  |  |
| Maternal schooling | 9.0 [9.0;11.5] | 9.0 [9.0;11.5] | 9.0 [9.0;11.5] | 9.0 [9.0;11.5] | 9.0 [9.0;11.5] | 9.0 [9.0;11.5] |  |  |
| Paternal schooling | 11.5 [9.0;11.5] | 11.5 [9.0;14.0] | 11.5 [9.0;14.0] | 11.5 [9.0;14.0] | 11.5 [9.0;14.0] | 11.5 [9.0;14.0] |  |  |
| Ethnicity |  |  |  |  |  |  |  |  |
| White | 207 (6.3%) | 205 (10.6%) | 206 (11.3%) | 204 (11.6%) | 204 (12.4%) | 205 (10.9%) |  |  |
| Black | 2568 (78.5%) | 1354 (70.2%) | 1229 (67.1%) | 1198 (68.0%) | 1096 (66.4%) | 1335 (71.0%) |  |  |
| Colored | 383 (11.7%) | 266 (13.8%) | 289 (15.8%) | 257 (14.6%) | 248 (15.0%) | 233 (12.4%) |  |  |
| Indian | 115 (3.5%) | 103 (5.3%) | 107 (5.8%) | 103 (5.8%) | 102 (6.2%) | 106 (5.6%) |  |  |
| Sex |  |  |  |  |  |  |  |  |
| Male | 1591 (48.6%) | 943 (48.9%) | 902 (49.3%) | 864 (49.0%) | 812 (49.2%) | 931 (49.5%) |  |  |
| Female | 1682 (51.4%) | 985 (51.1%) | 929 (50.7%) | 898 (51.0%) | 838 (50.8%) | 948 (50.5%) |  |  |

# Supplementary Table 3A. Loadings of harmonized index and cross-sectional indices with all assets for Pelotas 1993 cohort

|  | **Survey Year** | **Harmonized** | **1997** | **2004** | **2008** | **2011** | **2015** |  |  |  |
| --- | --- | --- | --- | --- | --- | --- | --- | --- | --- | --- |
|  | ***Variance explained by PC1 (%)*** | 44.6% | 55.7% | 50.2% | 43.2% | 42.3% | 30.6% |  |  |  |
|  | ***Correlation with harmonized*** | 1.00 | 0.94 | 0.95 | 0.96 | 0.94 | 0.78 |  |  |  |
| **Asset** | **Categorization** |  |  |  |  |  |  |  |  |  |
| Rooms per person | Crowding | 0.32 |  | 0.55 | 0.46 | 0.36 | 0.08 |  |  |  |
| Car | Yes, No | 0.74 | 0.8 | 0.81 | 0.77 | 0.75 | 0.67 |  |  |  |
| Computer | Yes, No | 0.81 |  | 0.86 | 0.83 | 0.8 | 0.73 |  |  |  |
| Duplex refrigerator | Yes, No | 0.67 |  | 0.75 | 0.67 | 0.59 | 0.65 |  |  |  |
| DVD player | Yes, No | 0.77 | 0.85 | 0.82 | 0.72 | 0.63 | 0.22 |  |  |  |
| Housekeeper | Yes, No | 0.63 | 0.74 | 0.77 | 0.82 | 0.81 |  |  |  |  |
| Radio | Yes, No | -0.19 | 0.64 | 0.47 | 0.4 |  | 0.26 |  |  |  |
| Refrigerator | Yes, No | 0.54 | 0.79 | 0.67 | 0.57 |  | -0.24 |  |  |  |
| Television | Yes, No | 0.84 | 0.85 | 0.77 |  |  |  |  |  |  |
| Vacuum cleaner | Yes, No | 0.77 | 0.82 | 0.83 | 0.81 | 0.77 |  |  |  |  |
| Washing machine | Yes, No | 0.77 | 0.8 | 0.78 | 0.77 | 0.69 | 0.74 |  |  |  |
| Piped water | Low, Medium, High | 0.62 |  |  |  |  |  |  |  |  |
| Housing material | Low, Medium, High |  | 0.66 | 0.66 |  |  |  |  |  |  |
| Toilet | Low, Medium, High |  | 0.78 |  |  |  |  |  |  |  |
| Motorcycle | Yes, No |  | 0.32 |  |  |  | 0.07 |  |  |  |
| Stereo | Yes, No |  |  | 0.7 | 0.67 |  |  |  |  |  |
| House ownership | Yes, No |  |  | 0.14 | 0.13 | 0.23 |  |  |  |  |
| Video game | Yes, No |  |  |  | 0.54 | 0.42 |  |  |  |  |
| Air conditioning | Yes, No |  |  |  |  | 0.76 | 0.83 |  |  |  |
| Desktop | Yes, No |  |  |  |  | 0.64 |  |  |  |  |
| Notebook computer | Yes, No |  |  |  |  | 0.68 |  |  |  |  |
| Microwave | Yes, No |  |  |  |  | 0.67 | 0.65 |  |  |  |
| Cleaning lady | Yes, No |  |  |  |  |  | 0.72 |  |  |  |
| Clothes dryer | Yes, No |  |  |  |  |  | 0.63 |  |  |  |
| Street is paved | Yes, No |  |  |  |  |  | 0.35 |  |  |  |

# Supplementary Table 3B. Loadings for harmonized index and cross-sectional indices with all assets for INCAP Longitudinal Study

|  | **Survey Year** | **Harmonized** | **1967^1^** | **1975** | **1987** | **1996** | **2002** | **2015-16** | **2017-18** |  |
| --- | --- | --- | --- | --- | --- | --- | --- | --- | --- | --- |
|  | ***Variance explained by PC1 (%)*** | 54.4% | 44.0% | 32.5% | 33.5% | 30.3% | 29.2% | 33.6% | 34.2% |  |
|  | ***Correlation with harmonized*** | 1.00 | 0.95 | 0.89 | 0.81 | 0.82 | 0.87 | 0.94 | 0.92 |  |
| **Asset** | **Categorization** |  |  |  |  |  |  |  |  |  |
| Crowding | Number of rooms/person | 0.44 | 0.38 | 0.24 | 0.02 | 0.12 | 0.25 | 0.34 | 0.31 |  |
| Bicycle | Yes, No | 0.59 |  |  | 0.55 | 0.45 | 0.47 | 0.26 | 0.18 |  |
| Car | Yes, No | 0.81 |  |  |  |  | 0.63 | 0.74 | 0.74 |  |
| Electricity | Yes, No | 0.94 |  | -0.08 | 0.69 | 0.52 |  |  |  |  |
| Motorcycle | Yes, No | 0.72 |  |  |  |  |  | 0.46 | 0.41 |  |
| House ownership | Yes, No | 0.09 | 0.07 | 0.29 | 0.57 | 0.51 | 0.43 | 0.07 | 0.11 |  |
| Radio | Yes, No | -0.17 | 0.4 | 0.55 | 0.68 | 0.79 | 0.04 | 0 | -0.01 |  |
| Refrigerator | Yes, No | 0.9 |  |  |  | 0.72 | 0.86 | 0.82 | 0.78 |  |
| Sewing machine | Yes, No | 0.46 |  | 0.78 | 0.6 | 0.6 | 0.62 | 0.49 | 0.45 |  |
| Television | Yes, No | 0.94 |  |  | 0.86 | 0.76 | 0.69 | 0.76 | 0.83 |  |
| Floor quality | Low, Medium, High | 0.87 |  | 0.56 | 0.68 | 0.63 | 0.59 | 0.68 | 0.73 |  |
| Kitchen location | Low, Medium, High | 0.68 | 0.75 | 0.69 | 0.33 | 0.17 | 0.33 | 0.55 | 0.51 |  |
| Roof quality | Low, Medium, High | 0.82 | 0.88 | 0.8 | 0.6 | 0.44 |  | 0.75 | 0.75 |  |
| Sewage facility | Low, Medium, High | 0.81 |  |  |  |  | 0.37 | 0.57 | 0.5 |  |
| Stove quality | Low, Medium, High | 0.84 |  | 0.78 |  | 0.63 |  | 0.81 | 0.82 |  |
| Toilet quality | Low, Medium, High | 0.80 | 0.63 | 0.19 |  |  | 0.45 | 0.41 | 0.37 |  |
| Wall quality | Low, Medium, High | 0.85 | 0.97 | 0.85 | 0.64 | 0.44 | 0.44 | 0.45 | 0.66 |  |
| Source of water quality | Low, Medium, High | 0.81 | 0.74 | 0.55 | 0.29 | 0.4 |  | 0.32 | 0.17 |  |
| Land ownership | Yes, No |  |  | 0.27 | 0.57 | 0.48 |  |  |  |  |
| Birds | Yes, No |  |  |  | 0.27 | 0.4 | 0.22 | -0.23 | -0.15 |  |
| Pigs | Yes, No |  |  |  | 0.27 | 0.25 | 0.13 | -0.19 | -0.1 |  |
| Turntable | Yes, No |  |  |  | 0.78 | 0.68 |  |  |  |  |
| Hand grinder | Yes, No |  |  |  | 0.49 | 0.31 |  |  |  |  |
| Electric iron | Yes, No |  |  |  | 0.78 | 0.79 | 0.78 |  |  |  |
| Cassette player | Yes, No |  |  |  |  | 0.74 | 0.15 |  |  |  |
| Sound system | Yes, No |  |  |  |  |  | 0.72 | 0.54 | 0.49 |  |
| Video player | Yes, No |  |  |  |  |  | 0.79 | 0.6 | 0.57 |  |
| Cable | Yes, No |  |  |  |  |  | 0.36 | 0.69 | 0.57 |  |
| Blender | Yes, No |  |  |  |  |  | 0.75 | 0.73 | 0.69 |  |
| Typewriter | Yes, No |  |  |  |  |  | 0.68 |  |  |  |
| Garbage disposal quality | Low, Medium, High |  |  |  |  |  |  | 0.59 |  |  |
| Microwave | Yes, No |  |  |  |  |  |  | 0.75 | 0.73 |  |
| Computer | Yes, No |  |  |  |  |  |  | 0.78 | 0.77 |  |
| Cellphone | Yes, No |  |  |  |  |  |  | 0.52 |  |  |
| Ipod | Yes, No |  |  |  |  |  |  | 0.53 | 0.68 |  |
| Washing machine | Yes, No |  |  |  |  |  |  | 0.83 | 0.79 |  |
| Internet | Yes, No |  |  |  |  |  |  |  | 0.84 |  |
| Direct TV | Yes, No |  |  |  |  |  |  |  | 0.61 |  |

1 Quality of roof, wall, kitchen, toilet and source of water were converted into binary variables (low vs medium) for 1967 due to absence of any values in ‘High’ category.

# Supplementary Table 3C. Loadings for harmonized index and cross-sectional indices with all assets for New Delhi Birth Cohort

|  | **Survey Year** | **Harmonized** | **1998-02** | **2006-09** | **2012-16** | **2016-19** |  |  |  |  |
| --- | --- | --- | --- | --- | --- | --- | --- | --- | --- | --- |
|  | ***Variance explained by PC1 (%)*** | 26.5% | 33.5% | 31.8% | 25.3% | 34.7% |  |  |  |  |
|  | ***Correlation with harmonized*** | 1.00 | 0.85 | 0.93 | 0.91 | 0.75 |  |  |  |  |
| **Asset** | **Categorization** |  |  |  |  |  |  |  |  |  |
| Crowding | Number of rooms per person | 0.29 | 0.39 | 0.41 | 0.4 | 0.31 |  |  |  |  |
| Air conditioner | Yes, No | 0.89 | 0.68 | 0.85 | 0.87 | 0.92 |  |  |  |  |
| Bicycle | Yes, No | 0.35 | -0.1 | 0.44 | 0.28 | 0.3 |  |  |  |  |
| Cable TV | Yes, No | -0.44 | 0.75 | -0.08 | -0.34 |  |  |  |  |  |
| Car | Yes, No | 0.75 | 0.76 | 0.85 | 0.85 | 0.84 |  |  |  |  |
| Cellphone | Yes, No | 0.81 |  |  |  |  |  |  |  |  |
| Computer | Yes, No | 0.89 | 0.62 | 0.81 | 0.82 | 0.89 |  |  |  |  |
| Cooler | Yes, No | -0.53 | 0.36 | -0.06 | -0.5 | -0.5 |  |  |  |  |
| Dish TV | Yes, No | 0.83 |  | 0.61 | 0.51 |  |  |  |  |  |
| Mixer grinder | Yes, No | 0.57 | 0.84 | 0.85 |  |  |  |  |  |  |
| Radio | Yes, No | -0.24 | 0.49 | 0.45 | 0.35 | 0.28 |  |  |  |  |
| Telephone | Yes, No | 0.01 | 0.82 | 0.66 | 0.71 | 0.53 |  |  |  |  |
| Television | Yes, No | 0.02 |  |  |  |  |  |  |  |  |
| Two wheeler | Yes, No | 0.03 | 0.52 | 0.2 | 0.09 | 0.04 |  |  |  |  |
| Washing machine | Yes, No | 0.66 | 0.76 | 0.73 | 0.63 |  |  |  |  |  |
| Drinking water source | Low, Medium, High | 0.12 | 0.3 |  | -0.02 |  |  |  |  |  |
| Sharing of drinking water | Low, Medium, High | -0.01 | 0.41 | 0.32 | 0.07 |  |  |  |  |  |
| General water | Low, Medium, High | 0.07 | 0.19 | 0.39 | 0.00 |  |  |  |  |  |
| Sharing of general water | Low, Medium, High | -0.14 | 0.55 | 0.28 | 0.00 |  |  |  |  |  |
| Sharing of toilet | Low, Medium, High | 0.53 | 0.59 |  |  |  |  |  |  |  |
| Television- color | Yes, No |  |  |  |  | -0.44 |  |  |  |  |
| Water pump | Yes, No |  |  |  |  | -0.27 |  |  |  |  |
| Sewing machine | Yes, No |  |  |  |  | -0.20 |  |  |  |  |
| Internet | Yes, No |  |  |  |  | 0.90 |  |  |  |  |
| Television - plasma | Yes, No |  |  |  |  | 0.78 |  |  |  |  |

# Supplementary Table 3D. Loadings for harmonized index and cross-sectional indices with all assets for Cebu Longitudinal Health and Nutrition Study

|  | **Survey Year** | **Harmonized** | **1983** | **1991** | **1994** | **1998** | **2002** | **2005** | **2009** | **2018** |
| --- | --- | --- | --- | --- | --- | --- | --- | --- | --- | --- |
|  | ***Variance explained by PC1 (%)*** | 35.5% | 29.7% | 40.1% | 40.7% | 39.8% | 33.5% | 36.2% | 42.9% | 34.5% |
|  | ***Correlation with harmonized*** | 1.00 | 0.92 | 0.99 | 0.98 | 0.98 | 0.96 | 0.96 | 0.93 | 0.94 |
| **Asset** | **Categorization** |  |  |  |  |  |  |  |  |  |
| Number of rooms per person | Crowding | 0.33 | 0.23 | 0.46 | 0.5 | 0.43 | 0.38 | 0.41 |  | 0.26 |
| Air conditioning | Yes, No | 0.8 |  |  |  |  |  | 0.85 | 0.89 | 0.87 |
| Bicycle | Yes, No | 0.36 | 0.32 | 0.4 | 0.39 | 0.34 | 0.32 | 0.26 | 0.23 | 0.28 |
| Car | Yes, No | 0.81 |  |  |  |  |  |  |  | 0.85 |
| Chicken/poultry | Yes, No | -0.18 | -0.11 | -0.27 | -0.24 | -0.23 | -0.18 | -0.14 |  | -0.1 |
| Electric fan | Yes, No | 0.78 | 0.9 | 0.9 | 0.88 | 0.86 |  | 0.8 | 0.82 | 0.65 |
| Electric iron | Yes, No | 0.87 | 0.84 | 0.86 | 0.84 | 0.83 | 0.82 | 0.8 | 0.79 | 0.79 |
| Electricity | Yes, No | 0.83 |  | 0.91 |  | 0.89 | 0.73 | 0.79 | 0.86 |  |
| Jepny | Yes, No | 0.65 |  |  |  |  | 0.62 | 0.62 | 0.69 | 0.52 |
| Living room set | Yes, No | 0.71 | 0.82 | 0.76 | 0.75 | 0.72 | 0.76 | 0.78 |  | 0.74 |
| Other appliances | Yes, No | 0.38 |  | 0.76 | 0.72 | 0.71 | 0.11 | 0.34 |  |  |
| House | Yes, No | 0.09 | -0.1 | -0.17 | -0.15 | -0.08 | 0.04 | 0.16 | 0.1 | 0.14 |
| Refrigerator | Yes, No | 0.88 | 0.92 | 0.88 | 0.87 | 0.88 | 0.85 | 0.85 | 0.85 | 0.83 |
| Cleanliness of are where food is stored | Low, Medium, High | 0.49 | 0.47 | 0.59 | 0.62 | 0.55 | 0.56 | 0.55 | 0.6 | 0.61 |
| Cooking fuel | Low, Medium, High | 0.82 | 0.67 | 0.8 | 0.83 | 0.82 | 0.82 | 0.78 | 0.8 | 0.61 |
| Garbage disposal | Low, Medium, High | 0.36 | 0.3 | -0.07 | -0.3 | -0.38 | 0.42 | 0.41 | 0.45 | 0.33 |
| Condition of area for excreta | Low, Medium, High | 0.25 | 0.31 | 0.35 | 0.39 | 0.37 | 0.35 | 0.31 | 0.33 | 0.39 |
| Lighting | Low, Medium, High | 0.91 | 0.83 | 0.92 |  | 0.87 | 0.76 | 0.7 | 0.96 |  |
| Housing material | Low, Medium, High | 0.66 | 0.7 | 0.7 | 0.75 | 0.7 | 0.67 | 0.7 | 0.74 | 0.73 |
| Neighborhood excreta removal | Low, Medium, High | 0.56 |  | 0.27 | 0.33 | 0.32 | 0.25 | 0.27 | 0.27 | 0.38 |
| Neighborhood garbage removal | Low, Medium, High | 0.61 |  | 0.42 | 0.38 | 0.46 | 0.39 | 0.27 | 0.38 | 0.42 |
| Drinking water | Low, Medium, High | 0.53 | 0.69 | 0.55 | 0.51 | 0.53 | 0.44 | 0.46 | -0.03 |  |
| Toilet | Low, Medium, High | 0.79 | 0.68 | 0.77 | 0.78 | 0.77 | 0.74 | 0.72 | 0.79 | 0.84 |
| Beds | Yes, No | 0.7 | 0.74 | 0.68 | 0.7 | 0.65 | 0.62 | 0.68 |  | 0.56 |
| Boat | Yes, No | -0.02 |  |  |  |  |  |  |  |  |
| Cattle (cows or carabaos) | Yes, No | -0.32 | -0.29 | -0.38 | -0.37 | -0.43 | -0.24 |  |  |  |
| Farm animals (goat, horse, pig etc) | Yes, No | -0.31 | -0.06 | -0.3 | -0.26 | -0.23 | -0.09 | -0.14 |  | 0.01 |
| Other vehicles (banca, motorcycle or tricycle with side-car etc) | Yes, No | 0.18 | 0.22 |  |  |  | 0.11 | 0.1 | 0.09 | -0.09 |
| Truck or bus | Yes, No | 0.52 |  |  |  |  |  |  |  |  |
| Television | Yes, No | 0.75 | 0.89 | 0.89 | 0.82 | 0.8 | 0.53 | 0.46 | 0.51 | 0.12 |
| Drinking water storage | Low, Medium, High |  | 0.37 |  |  |  |  |  |  |  |
| Beautician kit | Yes, No |  | 0.43 |  |  |  |  |  |  |  |
| Benches or chairs | Yes, No |  | 0.48 | 0.24 |  |  |  |  |  |  |
| Bottle brush | Yes, No |  | 0.53 |  |  |  |  |  |  |  |
| Chest/closet of drawers | Yes, No |  | 0.45 | 0.67 | 0.63 | 0.55 |  |  |  |  |
| Clay pots/ pan | Yes, No |  | -0.17 |  |  |  |  |  |  |  |
| Clay stove | Yes, No |  | 0.04 |  |  |  |  |  |  |  |
| Dining set | Yes, No |  | 0.77 | 0.76 |  |  | 0.62 | 0.69 |  | 0.68 |
| Feeding bottles | Yes, No |  | 0.34 |  |  |  |  |  |  |  |
| Flat iron | Yes, No |  | 0.51 |  |  |  |  |  |  |  |
| Glassware | Yes, No |  | 0.23 |  |  |  |  |  |  |  |
| Kerosene stove | Yes, No |  | 0.43 |  |  |  | -0.21 | -0.08 | 0.13 |  |
| Measuring spoon | Yes, No |  | 0.68 |  |  |  |  |  |  |  |
| Other business machine | Yes, No |  | 0.18 |  |  |  |  |  |  |  |
| Other kitchen equipment | Yes, No |  | 0.09 |  |  |  |  |  |  |  |
| Other agricultural equipment | Yes, No |  | -0.27 |  |  |  |  |  |  |  |
| Radio | Yes, No |  | 0.13 |  |  |  |  |  |  |  |
| Sewing machine | Yes, No |  | 0.6 |  |  |  | 0.48 | 0.51 | 0.52 | 0.5 |
| Tables | Yes, No |  | 0.55 | 0.31 |  |  |  |  |  |  |
| Tape recorder/ stereo set | Yes, No |  | 0.76 | 0.81 |  |  |  | 0.32 | 0.45 | 0.21 |
| Thermos bottle | Yes, No |  | 0.73 |  |  |  |  |  |  |  |
| Other house | Yes, No |  | 0.15 |  |  |  |  |  |  |  |
| Gas stove | Yes, No |  | 0.86 |  |  |  | 0.56 | 0.62 | 0.76 | 0.48 |
| Other furniture | Yes, No |  |  | 0.39 | 0.75 | 0.46 | 0.06 | 0.24 |  |  |
| Electricity in neighborhood | Yes, No |  |  | 0.77 | 0.73 | 0.77 |  |  |  |  |
| Neighborhood construction material | Low, Medium, High |  |  |  | 0.6 | 0.6 | 0.61 | 0.61 | 0.71 | 0.64 |
| China cabinet | Yes, No |  |  |  | 0.62 | 0.59 | 0.63 | 0.66 |  | 0.6 |
| Motorcycle | Yes, No |  |  |  | 0.56 | 0.5 | 0.48 | 0.46 | 0.45 | 0.41 |
| Digital camera | Yes, No |  |  |  | 0.87 | 0.82 | 0.37 | 0.67 | 0.8 | 0.72 |
| Phone | Yes, No |  |  |  | 0.86 | 0.88 | 0.85 | 0.77 | 0.28 | 0.48 |
| House is neat | Low, Medium, High |  |  |  |  | 0.53 | 0.54 | 0.5 | 0.57 | 0.55 |
| Cable + TV | Yes, No |  |  |  |  |  | 0.57 | 0.58 | 0.67 | 0.65 |
| CD player | Yes, No |  |  |  |  |  | 0.68 | 0.62 | 0.73 | 0.4 |
| Karaoke machine | Yes, No |  |  |  |  |  | 0.5 | 0.51 | 0.65 | 0.5 |
| Oven | Yes, No |  |  |  |  |  | 0.69 | 0.75 | 0.8 | 0.77 |
| Pressure cooker | Yes, No |  |  |  |  |  | 0.8 | 0.85 | 0.81 | 0.76 |
| Rice cooker | Yes, No |  |  |  |  |  | 0.77 | 0.76 | 0.72 | 0.64 |
| VCR player | Yes, No |  |  |  |  |  | 0.8 | 0.73 | 0.79 |  |
| Washing machine | Yes, No |  |  |  |  |  | 0.79 | 0.76 | 0.78 | 0.7 |
| Computer | Yes, No |  |  |  |  |  | 0.87 | 0.83 | 0.87 | 0.82 |
| Microwave | Yes, No |  |  |  |  |  |  | 0.79 | 0.8 |  |
| Video game | Yes, No |  |  |  |  |  |  | 0.64 | 0.75 | 0.75 |
| Non-drinking water source | Low, Medium, High |  |  |  |  |  |  |  |  | 0.42 |
| Vacuum cleaner | Yes, No |  |  |  |  |  |  |  |  | 0.8 |
| Tablet | Yes, No |  |  |  |  |  |  |  |  | 0.67 |
| Electric water dispenser | Yes, No |  |  |  |  |  |  |  |  | 0.65 |

# Supplementary Table 3E. Loadings for harmonized index and cross-sectional indices with all items for Birth to Twenty plus cohort

| **Asset** | **Categorization** | **Harmonized** | **1990-92** | **1997-98** | **2002-03** | **2006-07** | **2012-13** | **2017-18** |
| --- | --- | --- | --- | --- | --- | --- | --- | --- |
|  | ***Variance explained by PC1 (%)*** | 48.4% | 41.2% | 48.1% | 42.0% | 39.8% | 60.6% | 38.0% |
|  | ***Correlation with harmonized*** | 1.00 | 0.93 | 0.96 | 0.95 | 0.93 | 0.84 | 0.90 |
| Car | Yes, No | 0.65 | 0.69 | 0.64 | 0.61 | 0.72 | 0.67 | 0.68 |
| Electricity | Yes, No | 0.69 | 0.64 |  |  |  |  |  |
| Microwave | Yes, No | 0.76 |  | 0.71 | 0.66 | 0.74 | 0.86 | 0.83 |
| Radio | Yes, No | 0.48 | 0.33 | 0.41 | 0.5 | 0.48 | 0.77 |  |
| Refrigerator | Yes, No | 0.87 | 0.77 | 0.75 | 0.74 | 0.71 | 0.96 | 0.81 |
| Telephone | Yes, No | 0.43 | 0.64 | 0.66 | 0.57 | 0.57 | 0.58 | 0.42 |
| Television | Yes, No | 0.81 | 0.7 | 0.7 | 0.64 |  | 0.95 | 0.72 |
| Washing machine | Yes, No | 0.83 | 0.81 | 0.76 | 0.75 | 0.77 | 0.78 | 0.75 |
| Indoor flush toilet | Yes, No | 0.66 | 0.68 | 0.77 | 0.71 | 0.71 |  | 0.63 |
| Indoor hot/cold water | Yes, No | 0.64 | 0.74 | 0.79 | 0.77 | 0.63 |  | 0.66 |
| Housing type | Low, Medium, High |  | 0.33 | 0.71 | 0.66 |  |  | 0.61 |
| House ownership | Yes, No |  | 0.56 | 0.65 |  |  |  | 0.28 |
| Solo usage of water | Yes, No |  | 0.72 |  | 0.6 | 0.03 |  |  |
| Solo usage of toilet | Yes, No |  | 0.68 |  | 0.5 | -0.39 |  |  |
| DVD player | Yes, No |  |  | 0.7 | 0.75 | 0.73 | 0.84 |  |
| Cellphone | Yes, No |  |  |  | 0.5 | 0.36 |  | 0.5 |
| Mnet | Yes, No |  |  |  |  | 0.70 |  |  |
| Satellite TV | Yes, No |  |  |  |  | 0.72 |  |  |
| Computer | Yes, No |  |  |  |  | 0.72 | 0.69 | 0.65 |
| Internet | Yes, No |  |  |  |  |  | 0.58 | 0.54 |
| Number of rooms per person | Crowding |  | 0.47 |  |  |  |  | 0.09 |

# Supplementary Table 4. Tucker index of congruence between harmonized index and cross-sectional asset indices created using same set of covariates

|  |  | **Pelotas 1993**  **(Brazil)** |  | **INCAP**  **(Guatemala)** |  | **NDBC**  **(India)** |  | **CLHNS**  **(Philippines)** |  | **Birth to Twenty plus (South Africa)** |
| --- | --- | --- | --- | --- | --- | --- | --- | --- | --- | --- |
|  | **Age at wave** | **phi** | **Age at wave** | **phi** | **Age at wave** | **phi** | **Age at wave** | **phi** | **Age at wave** | **phi** |
| 1 | 3-4 | 0.92 | 0-5 | 0.40 | 27-33 | 0.52 | 0 | 0.98 | 0-2 | 0.99 |
| 2 | 11-12 | 0.96 | 0-7 | 0.81 | 34-40 | 0.81 | 7-8 | 0.97 | 7-8 | 0.99 |
| 3 | 13-14 | 0.96 | 10-25 | 0.91 | 40-47 | 0.87 | 12-13 | 0.96 | 12-13 | 0.99 |
| 4 | 18 | 0.99 | 19-34 | 0.91 | 44-51 | 0.83 | 15-16 | 0.95 | 16-17 | 0.99 |
| 5 | 22 | 0.75 | 25-40 | 0.96 |  |  | 18-19 | 0.98 | 22-23 | 0.95 |
| 6 |  |  | 37-55 | 0.97 |  |  | 21-22 | 0.98 | 27-28 | 0.99 |
| 7 |  |  | 40-57 | 0.96 |  |  | 25-26 | 0.97 |  |  |
| 8 |  |  |  |  |  |  | 33-36 | 0.96 |  |  |

# Supplementary Table 5A. Loadings for harmonized index and cross-sectional indices with same assets as harmonized index for INCAP Longitudinal Study by Urban and Rural strata

|  |  | **Harmonized** | **2015-16** |  | **2017-18** |  |  |  |  |  |
| --- | --- | --- | --- | --- | --- | --- | --- | --- | --- | --- |
|  | ***Strata*** |  | Urban | Rural | Urban | Rural |  |  |  |  |
|  | ***Variance explained by PC1 (%)*** | 54.4% | 35.5% | 30.6% | 37.0% | 31.8% |  |  |  |  |
|  | ***Correlation with harmonized*** |  | 0.98 | 0.97 | 0.96 | 0.95 |  |  |  |  |
| **Asset** | **Categorization** |  |  |  |  |  |  |  |  |  |
| Crowding | Number of rooms/person | 0.44 | 0.47 | 0.4 | 0.38 | 0.37 |  |  |  |  |
| Bicycle | Yes, No | 0.59 | 0.29 | 0.09 | 0.18 | 0.15 |  |  |  |  |
| Car | Yes, No | 0.81 | 0.72 | 0.68 | 0.72 | 0.74 |  |  |  |  |
| Electricity | Yes, No | 0.94 |  |  |  |  |  |  |  |  |
| Motorcycle | Yes, No | 0.72 | 0.39 | 0.14 | 0.57 | 0.59 |  |  |  |  |
| House ownership | Yes, No | 0.09 | 0.23 | 0.15 | 0.26 | 0.33 |  |  |  |  |
| Radio | Yes, No | -0.17 | 0.04 | -0.05 | 0.04 | 0.02 |  |  |  |  |
| Refrigerator | Yes, No | 0.9 | 0.76 | 0.73 | 0.79 | 0.77 |  |  |  |  |
| Sewing machine | Yes, No | 0.46 | 0.47 | 0.4 | 0.56 | 0.55 |  |  |  |  |
| Television | Yes, No | 0.94 |  | 0.75 | 0.71 | 0.8 |  |  |  |  |
| Floor quality | Low, Medium, High | 0.87 | 0.73 | 0.76 | 0.75 | 0.77 |  |  |  |  |
| Kitchen location | Low, Medium, High | 0.68 | 0.7 | 0.66 | 0.56 | 0.49 |  |  |  |  |
| Roof quality | Low, Medium, High | 0.82 | 0.77 | 0.84 | 0.74 | 0.76 |  |  |  |  |
| Sewage facility | Low, Medium, High | 0.81 | 0.75 | 0.76 | 0.4 | 0.3 |  |  |  |  |
| Stove quality | Low, Medium, High | 0.84 | 0.84 | 0.87 | 0.78 | 0.75 |  |  |  |  |
| Toilet quality | Low, Medium, High | 0.8 | 0.69 | 0.69 | 0.23 | 0.15 |  |  |  |  |
| Wall quality | Low, Medium, High | 0.85 | 0.38 | 0.77 | 0.63 | 0.74 |  |  |  |  |
| Source of water quality | Low, Medium, High | 0.81 |  | 0.45 | 0.21 | 0.18 |  |  |  |  |

1 Quality of roof, wall, kitchen, toilet and source of water were converted into binary variables (low vs medium) for 1967 due to absence of any values in ‘High’ category.

# Supplementary Table 5B. Loadings for harmonized index and cross-sectional indices with same assets as harmonized index for Cebu Longitudinal Health and Nutrition Study for Rural strata

|  |  | **Harmonized** | **1983** | **1991** | **1994** | **1998** | **2002** | **2005** | **2009** | **2018** |
| --- | --- | --- | --- | --- | --- | --- | --- | --- | --- | --- |
|  | ***Variance explained by PC1 (%)*** | 35.5% | 24.0% | 40.6% | 39.2% | 40.4% | 31.0% | 34.3% | 35.4% | 35.1% |
|  | ***Correlation with harmonized*** | 1.00 | 0.97 | 0.99 | 0.95 | 0.99 | 0.99 | 0.99 | 0.99 | 0.99 |
| **Asset** | **Categorization** |  |  |  |  |  |  |  |  |  |
| Number of rooms per person | Crowding | 0.33 | 0.3 | 0.53 | 0.53 | 0.46 | 0.38 | 0.36 | 0.35 | 0.37 |
| Air conditioning | Yes, No | 0.8 |  |  |  |  |  |  | 0.9 | 0.93 |
| Bicycle | Yes, No | 0.36 | 0.42 | 0.48 | 0.43 | 0.38 | 0.43 | 0.33 | 0.33 | 0.39 |
| Car | Yes, No | 0.81 |  |  |  |  |  |  |  | 0.91 |
| Chicken/poultry | Yes, No | -0.18 | -0.36 | -0.33 | -0.32 | -0.29 | -0.33 | -0.25 | -0.18 | -0.43 |
| Electric fan | Yes, No | 0.78 |  | 0.91 | 0.88 | 0.9 |  | 0.86 | 0.83 | 0.79 |
| Electric iron | Yes, No | 0.87 |  | 0.89 | 0.85 | 0.84 | 0.83 | 0.82 | 0.78 | 0.74 |
| Electricity | Yes, No | 0.83 |  | 0.89 |  | 0.87 | 0.84 | 0.82 | 0.81 |  |
| Jepny | Yes, No | 0.65 |  |  |  |  |  | 0.67 | 0.82 | 0.6 |
| Living room set | Yes, No | 0.71 | 0.64 | 0.77 | 0.7 | 0.69 | 0.7 | 0.74 | 0.55 | 0.66 |
| Other appliances | Yes, No | 0.38 |  |  | 0.65 | 0.68 | 0.08 |  |  |  |
| House | Yes, No | 0.09 | -0.31 |  |  |  | -0.06 | 0.18 | 0 | -0.05 |
| Refrigerator | Yes, No | 0.88 |  | 0.88 | 0.88 | 0.88 | 0.85 | 0.84 | 0.84 | 0.78 |
| Cleanliness of are where food is stored | Low, Medium, High | 0.49 | 0.4 | 0.54 | 0.65 | 0.57 | 0.42 | 0.45 | 0.6 | 0.48 |
| Cooking fuel | Low, Medium, High | 0.82 |  | 0.76 | 0.83 | 0.84 | 0.79 | 0.8 | 0.82 | 0.75 |
| Garbage disposal | Low, Medium, High | 0.36 | 0.16 | 0.25 | 0.09 | -0.18 | 0.48 | 0.51 | 0.47 | 0.35 |
| Condition of area for excreta | Low, Medium, High | 0.25 | 0.29 | 0.32 | 0.43 | 0.34 | 0.08 | 0.19 | 0.3 | 0.35 |
| Lighting | Low, Medium, High | 0.91 | 0.77 | 0.89 |  | 0.87 | 0.86 | 0.75 | 0.88 |  |
| Housing material | Low, Medium, High | 0.66 | 0.61 | 0.64 | 0.75 | 0.7 | 0.62 | 0.72 | 0.7 | 0.69 |
| Neighborhood excreta removal | Low, Medium, High | 0.56 |  | 0.21 | 0.41 | 0.24 | 0.02 | 0.16 | 0.23 | 0.31 |
| Neighborhood garbage removal | Low, Medium, High | 0.61 |  | 0.37 |  | 0.53 | 0.14 | 0.13 | 0.34 | 0.3 |
| Drinking water | Low, Medium, High | 0.53 | 0.72 | 0.53 | 0.42 | 0.55 | 0.59 | 0.52 | 0.2 | 0.8 |
| Toilet | Low, Medium, High | 0.79 | 0.77 | 0.76 | 0.75 | 0.81 | 0.83 | 0.8 | 0.87 | 0.92 |
| Beds | Yes, No | 0.7 | 0.57 | 0.54 | 0.69 | 0.57 | 0.58 | 0.74 | 0.53 | 0.5 |
| Boat | Yes, No | -0.02 |  |  |  |  |  |  |  |  |
| Cattle (cows or carabaos) | Yes, No | -0.32 | -0.43 | -0.32 | -0.33 | -0.42 | -0.31 | -0.25 | -0.22 | -0.41 |
| Farm animals (goat, horse, pig etc) | Yes, No | -0.31 | -0.31 | -0.28 | -0.26 | -0.23 | -0.23 | -0.24 | -0.23 | -0.28 |
| Other vehicles (banca, motorcycle or tricycle with side-car etc) | Yes, No | 0.18 | 0.03 |  |  |  | 0.33 | 0.31 | 0.31 |  |
| Truck or bus | Yes, No | 0.52 |  |  |  |  |  |  |  |  |
| Television | Yes, No | 0.75 |  | 0.89 | 0.8 | 0.77 | 0.76 | 0.71 | 0.65 | 0.15 |

# Supplementary Table 5C. Loadings for harmonized index and cross-sectional indices with same assets as harmonized index for Cebu Longitudinal Health and Nutrition Study for Urban strata

|  |  | **Harmonized** | **1983** | **1991** | **1994** | **1998** | **2002** | **2005** | **2009** | **2018** |
| --- | --- | --- | --- | --- | --- | --- | --- | --- | --- | --- |
|  | ***Variance explained by PC1 (%)*** | 35.5% | 38.4% | 41.4% | 40.2% | 39.6% | 31.6% | 32.4% | 34.1% | 35.4% |
|  | ***Correlation with harmonized*** | 1.00 | 0.98 | 0.99 | 0.99 | 0.98 | 0.99 | 0.99 | 0.98 | 0.99 |
| **Asset** | **Categorization** |  |  |  |  |  |  |  |  |  |
| Number of rooms per person | Crowding | 0.33 | 0.29 | 0.51 | 0.53 | 0.48 | 0.34 | 0.43 | 0.41 | 0.24 |
| Air conditioning | Yes, No | 0.8 |  |  |  |  | 0.85 | 0.86 | 0.88 | 0.86 |
| Bicycle | Yes, No | 0.36 | 0.23 | 0.34 | 0.31 | 0.3 | 0.26 | 0.17 | 0.14 | 0.21 |
| Car | Yes, No | 0.81 |  |  |  |  |  | 0.81 | 0.89 | 0.86 |
| Chicken/poultry | Yes, No | -0.18 | 0.02 | -0.07 | -0.07 | -0.01 | -0.07 | -0.07 | -0.07 | 0.02 |
| Electric fan | Yes, No | 0.78 | 0.89 | 0.86 | 0.87 | 0.82 |  | 0.74 | 0.77 | 0.62 |
| Electric iron | Yes, No | 0.87 | 0.83 | 0.83 | 0.84 | 0.81 | 0.8 | 0.76 | 0.77 | 0.79 |
| Electricity | Yes, No | 0.83 |  | 0.87 | 0.87 | 0.88 | 0.7 |  |  |  |
| Jepny | Yes, No | 0.65 |  |  |  |  | 0.57 | 0.64 | 0.65 | 0.62 |
| Living room set | Yes, No | 0.71 | 0.81 | 0.79 | 0.81 | 0.77 | 0.76 | 0.78 | 0.63 | 0.73 |
| Other appliances | Yes, No | 0.38 |  | 0.81 | 0.77 | 0.75 | 0.18 | 0.25 |  |  |
| House | Yes, No | 0.09 | -0.01 | -0.01 | 0 | 0.07 | 0.09 | 0.17 | 0.13 | 0.2 |
| Refrigerator | Yes, No | 0.88 | 0.93 | 0.88 | 0.86 | 0.89 | 0.81 | 0.83 | 0.85 | 0.87 |
| Cleanliness of are where food is stored | Low, Medium, High | 0.49 | 0.53 | 0.68 | 0.68 | 0.62 | 0.66 | 0.63 | 0.66 | 0.67 |
| Cooking fuel | Low, Medium, High | 0.82 | 0.67 | 0.74 | 0.77 | 0.76 | 0.78 | 0.76 | 0.79 | 0.56 |
| Garbage disposal | Low, Medium, High | 0.36 | 0.28 | -0.01 | -0.25 | -0.27 | 0.38 | 0.37 | 0.42 | 0.35 |
| Condition of area for excreta | Low, Medium, High | 0.25 | 0.37 | 0.54 | 0.47 | 0.51 | 0.55 | 0.49 | 0.49 | 0.49 |
| Lighting | Low, Medium, High | 0.91 | 0.83 | 0.86 | 0.87 | 0.85 | 0.74 |  |  | 0.91 |
| Housing material | Low, Medium, High | 0.66 | 0.72 | 0.78 | 0.74 | 0.72 | 0.72 | 0.67 | 0.76 | 0.76 |
| Neighborhood excreta removal | Low, Medium, High | 0.56 |  | 0.5 | 0.42 | 0.5 | 0.44 | 0.48 | 0.46 | 0.5 |
| Neighborhood garbage removal | Low, Medium, High | 0.61 |  | 0.57 | 0.41 | 0.55 | 0.55 | 0.49 | 0.53 | 0.5 |
| Drinking water | Low, Medium, High | 0.53 | 0.71 | 0.22 | 0.34 | 0.31 | 0.37 | 0.37 | -0.18 |  |
| Toilet | Low, Medium, High | 0.79 | 0.66 | 0.66 | 0.64 | 0.64 | 0.67 | 0.74 | 0.78 |  |
| Beds | Yes, No | 0.7 | 0.72 | 0.69 | 0.67 | 0.66 | 0.58 | 0.67 | 0.55 | 0.58 |
| Boat | Yes, No | -0.02 |  |  |  |  |  |  |  |  |
| Cattle (cows or carabaos) | Yes, No | -0.32 |  |  |  |  |  |  |  |  |
| Farm animals (goat, horse, pig etc) | Yes, No | -0.31 | 0.11 | -0.05 | 0.02 | 0.01 | 0.04 | 0.02 | 0 |  |
| Other vehicles (banca, motorcycle or tricycle with side-car etc) | Yes, No | 0.18 | 0.31 |  |  |  | 0.04 | 0.04 | 0.02 | -0.13 |
| Truck or bus | Yes, No | 0.52 |  |  |  |  |  |  |  |  |
| Television | Yes, No | 0.75 | 0.89 | 0.85 | 0.83 | 0.82 | 0.5 | 0.41 | 0.41 | 0.3 |

# Supplementary Table 6. Correlation of schooling and health measures with cross-sectional asset index in corresponding wave among those who participated in adulthood

|  |  | **Pelotas 1993**  **(Brazil)** |  | **INCAP**  **(Guatemala)** |  | **NDBC**  **(India)** |  | **CLHNS**  **(Philippines)** |  | **Birth to Twenty plus (South Africa)** |
| --- | --- | --- | --- | --- | --- | --- | --- | --- | --- | --- |
|  | **Age at wave** | **rho** | **Age at wave** | **rho** | **Age at wave** | **rho** | **Age at wave** | **rho** | **Age at wave** | **rho** |
| **Schooling** | | | | | | | | | | |
| 1 | 3-4^a^ | 0.55 | 0-7 ^a^ | 0.14 | 0-2^b^ | Not available | 0^a^ | 0.58 | 0-2^a^ | 0.27 |
| 2 | 11-12^a^ | 0.60 | 10-25 | 0.31 | 27-33 | 0.48 | 7-8^a^ | 0.56 | 7-8^a^ | 0.30 |
| 3 | 13-14^a^ | 0.57 | 19-34 | 0.3 | 34-40 | 0.48 | 12-13^a^ | 0.56 | 12-13^a^ | 0.29 |
| 4 | 18 | 0.47 | 25-40 | 0.37 | 40-47 | 0.44 | 15-16 | 0.50 | 16-17 | 0.19 |
| 5 | 22 | 0.44 | 37-55 | 0.5 | 44-51 | 0.52 | 18-19 | 0.53 | 22-23 | 0.21 |
| 6 |  |  | 40-57 | 0.5 |  |  | 21-22 | 0.53 | 27-28 | 0.30 |
| 7 |  |  |  |  |  |  | 25-26 | 0.54 |  |  |
| 8 |  |  |  |  |  |  | 33-36 | 0.56 |  |  |
| **HAZ at 2y** | | | | | | | | | | |
| 9 | 2^b^ | Not available | 2 | 0.10 | 2^b^ | Not available | 2 | 0.25 | 2 | 0.12 |
| **BMI in adulthood** | | | | | | | | | | |
| 10 | 22 | -0.05 | 37-55 | 0.18 | 44-51 | 0.23 | 33-36 | 0.19 | 22-23 | 0.04 |

Sample sizes among those who participated in adulthood varied for above Pearson correlations: Brazil (995;3608;3576;3519;3805;3559), Guatemala (1346;931;641;821;1160;1265;723;1143), India (868;807;790;841;828), Philippines (1326; 1321; 1325; 1325; 1303; 1311; 1274; 1249; 1326; 1285;1304), and South Africa (1132;999;1071;1201; 1274; 1393; 856; 1202). This is not the sample size of participants at each wave (non-monotone missingness).

a Correlation with maternal schooling. Values from 1967 and 1975 were combined for Guatemala (n = 2392).

b Temporally harmonized asset index was not available in childhood for NDBC and before 3 years for Pelotas 1993

# Supplementary Table 7. Correlation of harmonized index with alternate factor extraction procedures

| **Factor extraction** | **Correlation matrix** | **Pelotas 1993**  **(Brazil)** | **INCAP**  **(Guatemala)** | **NDBC**  **(India)** | **CLHNS**  **(Philippines)** | **Birth to Twenty plus (South Africa)** |
| --- | --- | --- | --- | --- | --- | --- |
| Exploratory Factor Analysis | Polychoric | 0.99 | 0.95 | 0.99 | 0.96 | 0.99 |
| Exploratory Factor Analysis | Pearson | 0.99 | 0.94 | 0.99 | 0.94 | 0.99 |
| Principal Component Analysis | Pearson | 1.00 | 0.99 | 1.00 | 0.99 | 1.00 |
| Multiple Correspondence Analysis |  | 0.99 | 0.98 | 0.99 | 0.99 | 1.00 |
